# Disruption of amino acid metabolism in acute cerebrovascular diseases: diagnostic and pathobiochemical perspectives

**DOI:** 10.3389/fneur.2026.1876777

**Published:** 2026-09-07

**Authors:** Sharbanu Battakova, Marina Grigolashvili, Dmitriy Klyuyev, Dmitry Kasatkin, Yelena Shayakhmetova, Mira Beisembayeva, Shynar Muratbekova, Alina Koshelyuk, Alexandr Marchenko

**Affiliations:** 1 Department of Neurology, Psychiatry and Rehabilitation, Karaganda Medical University, Karaganda, Kazakhstan; 2 Institute of Life Sciences, Karaganda Medical University, Karaganda, Kazakhstan; 3 Department of Neurology, Yaroslavl State Medical University, Yaroslavl, Russia

**Keywords:** acute cerebrovascular diseases, acute ischemic stroke, amino acid metabolism, diagnostic biomarkers, hemorrhagic stroke, plasma amino acids, stroke metabolomics, transient ischemic attack

## Abstract

**Introduction:**

Early differential diagnosis of acute cerebrovascular events remains a key clinical challenge, particularly during the hyperacute phase. This study aimed to assess plasma amino acid profiles and their dynamic changes as potential biomarkers and to evaluate their contribution to diagnostic accuracy.

**Methods:**

This prospective observational clinical and laboratory study included 297 participants: 122 patients with acute ischemic stroke (AIS), 50 with hemorrhagic stroke (HS), 50 with transient ischemic attack (TIA), and 75 control participants. Plasma concentrations of six amino acids were determined using high-performance liquid chromatography coupled with mass spectrometry (HPLC-MS). Patients with acute cerebrovascular disease were sampled at 3-4.5 h and 12 h after symptom onset, whereas control participants provided a baseline sample only. Statistical analyses included non-parametric tests, correlation analysis, ROC curve analysis, and multivariable logistic regression.

**Results:**

Significant between-group differences in amino acid concentrations were observed, particularly during the hyperacute phase. Individual amino acids showed limited discriminative ability when assessed in isolation (AUC approximately 0.61-0.65). Analysis of temporal changes identified additional subtype-related metabolic patterns, particularly in the differentiation of AIS and TIA. Addition of metabolomic variables to the clinical model increased the AUC from 0.734 to 0.803 (DeLong *p* = 0.007).

**Discussion:**

Plasma amino acid profiles are associated with systemic metabolic alterations during acute cerebrovascular events. Although individual metabolites have limited diagnostic utility as standalone biomarkers, their integration with clinical variables and assessment of temporal changes may provide complementary information for early diagnostic differentiation.

## 1 Introduction

Acute cerebrovascular disorders, including acute ischemic stroke (AIS), hemorrhagic stroke (HS), and transient ischemic attack (TIA) continue to be among the leading causes of mortality and morbidity globally (1, 2). Early diagnosis and differentiation of various types of acute cerebrovascular disorders are essential for determining the appropriate treatment strategy, particularly in situations where there is a limited therapeutic window available (3).

Despite the widespread use of neuroimaging techniques, the diagnostic capabilities in the hyperacute phase of disease remain limited (4, 5). In the hyperacute phase after the onset of acute neurological symptoms, CT scans may not be sensitive to ischemic damage, and the clinical presentation does not always provide a reliable means of differentiating between AIS, HS, and TIA. Therefore, there is a need for readily available and informative biomarkers that could supplement existing diagnostic techniques.

Acute ischemic brain damage is associated with severe metabolic disturbances, including dysregulation of amino acid metabolism. Glutamic acid has been shown to be involved in mechanisms of excitotoxicity (6), phenylalanine may represent a systemic catabolic response and inflammatory process (7–9), and changes in tryptophan metabolism have been linked to the activation of the kynurenine pathway (10). Therefore, the amino acid profile of plasma in the hyperacute phase of the disease may already reflect both local neurochemical processes and the body’s systemic response to acute injury.

Despite the increasing interest in metabolic research, the clinical significance of amino acid analysis in the early diagnosis of various forms of acute cerebrovascular disease remains poorly understood. Specifically, data comparing patients with AIS, HS, TIA, and control groups at the hyperacute phase of disease are limited.

The aim of this research is to assess changes in the amino acid composition of plasma in patients with acute cerebrovascular disorders and to analyze their correlation with clinical and laboratory parameters.

## 2 Materials and methods

### 2.1 Research design and population

This prospective observational clinical and laboratory study included 297 participants: 122 patients with acute ischemic stroke (AIS), 50 with hemorrhagic stroke (HS), 50 with transient ischemic attack (TIA), and 75 control subjects recruited at two stroke centers in the Karaganda Region, Republic of Kazakhstan. The study was conducted between January 2024 and June 2025.

The final diagnosis of AIS, HS, or TIA was established by two experienced stroke neurologists according to current clinical guidelines, based on clinical presentation, repeated neurological examinations, neuroimaging findings, and 24-h clinical follow-up. Because neuroimaging performed within the first 3–4.5 h after symptom onset may not always allow reliable differentiation between AIS, HS, and TIA, study group allocation was performed in two sequential stages.

At enrollment, all consecutive patients presenting within 4.5 h after symptom onset with suspected acute cerebrovascular disease underwent a standardized neurological examination and emergency neuroimaging according to the institutional stroke protocol. Based on these findings, a preliminary diagnosis of AIS, HS, or TIA was assigned prospectively, and the first blood sample was collected before the final diagnosis was established.

Within the first 24 h after admission, the final diagnosis was established using all available clinical information, repeated neurological examinations, and follow-up CT and/or MRI findings. Patients whose initial diagnosis was revised during this diagnostic adjudication process were reassigned to the appropriate study group or excluded if the final diagnosis did not meet the predefined eligibility criteria.

Metabolomic analyses were performed only after completion of clinical follow-up and final diagnostic classification. Therefore, amino acid measurements were unavailable during diagnostic adjudication and could not influence the assignment of patients to the study groups.

The final study cohort comprised hospitalized patients as well as patients managed in outpatient or day-hospital settings (primarily patients with TIA), provided that all predefined eligibility criteria were fulfilled.

To improve the clinical homogeneity of the study population and reduce variability associated with extremely mild and very severe stroke presentations, enrollment in the stroke groups was restricted *a priori* to patients with NIHSS scores between 3 and 22, as specified in the study protocol.

The inclusion criteria common to all stroke groups (AIS, HS, and TIA) were: (1) age ≥18 years; (2) NIHSS score between 3 and 22; (3) presentation within 3–4.5 h after symptom onset; and (4) written informed consent provided by the patient or a legally authorized representative.

Additional diagnostic criteria for the AIS group included the absence of intracranial hemorrhage or brain tumor on the initial CT and/or MRI examination, with the final diagnosis confirmed by clinical evaluation and follow-up neuroimaging within the first 24 h after admission.

For the HS group, inclusion required neuroimaging confirmation of intracranial hemorrhage and the absence of imaging findings consistent with AIS or brain tumor. The diagnosis was confirmed based on clinical assessment and follow-up neuroimaging within the first 24 h after admission.

For the TIA group, inclusion required the absence of intracranial hemorrhage, acute cerebral infarction, or brain tumor on CT and/or MRI at admission and during the first 24 h of follow-up, together with complete resolution of neurological symptoms consistent with current diagnostic criteria.

Control subjects were required to be ≥18 years of age, have grade III arterial hypertension, no focal neurological deficits, no history of malignancy, myocardial infarction, or other acute cardiovascular diseases, no acute illness or exacerbation of chronic disease at the time of enrollment, and to provide written informed consent. The control group was intentionally composed of participants with grade III arterial hypertension but without acute cerebrovascular disease in order to serve as a vascular risk-matched reference group. Because arterial hypertension is the major vascular risk factor for stroke and may itself influence amino acid metabolism, this design was intended to reduce confounding related to chronic hypertension and to facilitate the identification of metabolomic alterations specifically associated with acute cerebrovascular events rather than with underlying vascular disease. Control subjects were frequency matched to the stroke groups by age and sex.

The exclusion criteria for all study groups included malignancy, active cardiovascular inflammatory diseases (including subacute bacterial endocarditis and acute pericarditis), gastrointestinal bleeding, severe hepatic dysfunction, end-stage renal disease, and any other condition considered likely to influence the laboratory parameters under investigation. Participants could withdraw from the study at any time without affecting their medical care.

The study protocol was approved by the institutional Ethics Committee (Approval No. 19), and written informed consent was obtained from all participants or their legally authorized representatives before enrollment.

This observational study was reported in accordance with the Strengthening the Reporting of Observational Studies in Epidemiology (STROBE) statement. The diagnostic accuracy and prediction model analyses were additionally reported in accordance with the relevant principles of the Standards for Reporting Diagnostic Accuracy Studies (STARD 2015) and the Transparent Reporting of a Multivariable Prediction Model for Individual Prognosis or Diagnosis (TRIPOD) statement, where applicable.

### 2.2 Calculating the sample size

The sample size was determined to test the hypothesis of a difference in the amino acid profile between patients with AIS group and the control group, using phenylalanine levels as the primary variable. Based on an expected effect size of approximately 0.5 (Cohen’s *d*), *α* = 0.05, and a power of 80%, a minimum sample size of 63 participants in each group was required. The actual sample size (122 in the AIS group and 75 in the control group) provided adequate statistical power for the study. The HS and TIA groups were also included for secondary analyses, and multiple comparisons were adjusted using the Benjamini–Hochberg method to control for Type I error.

### 2.3 Clinical examination and data collection

All patients in the study groups underwent a thorough clinical examination, which included the collection of subjective complaints, medical and life history information from the patient and their relatives, as well as a detailed assessment of their neurological status. The neurological examination was conducted by a neurologist within the first few hours after admission to the stroke center using a standard clinical protocol.

The severity of the neurological impairment was assessed using the NIHSS upon admission. In addition, a retrospective review of the medical records was performed to collect demographic and clinical characteristics, medical history, routine laboratory data (including complete blood count, biochemical tests, and coagulation parameters), and neuroimaging findings (CT and/or MRI). Clinical and laboratory data analyzed in the present study were obtained at hospital admission as part of routine clinical care. Because clinical and routine laboratory data were collected retrospectively from medical records, the availability of individual laboratory variables depended on whether the corresponding tests had been requested as part of routine clinical care. Missing values therefore reflected differences in routine laboratory ordering rather than predefined study procedures. According to the study protocol, laboratory investigations were performed according to routine clinical practice rather than predefined study procedures, stroke subtype, or clinical severity. Consequently, analyses involving these variables were performed using available-case analysis without imputation of missing values. The number of valid observations for each variable is reported in the corresponding tables.

Although missing values reflected routine clinical practice rather than predefined study procedures, the mechanism of missingness was not formally evaluated. Therefore, analyses based on incomplete variables should be interpreted with caution.

### 2.4 Collection, storage and preparation of plasma samples

Blood sampling in all study groups was conducted under standardized conditions, in accordance with uniform requirements for the pre-analytical phase. The first sample was collected upon admission to the hospital, within the therapeutic window of 3–4.5 h following the onset of acute neurological symptoms. The second sample was taken 12 h following their onset. Paired EDTA plasma samples were available for all patients with acute cerebrovascular disease (AIS, *n* = 122; HS, *n* = 50; TIA, *n* = 50), whereas control participants (*n* = 75) provided a single baseline plasma sample only.

The choice of these specific time intervals for blood collection was based on the dynamic nature of metabolic disturbances during the acute phase of stroke and the need to compare early versus delayed changes in amino acid profiles of blood plasma. This allowed us to assess the diagnostic potential of the metabolites under investigation in different stages of the pathological process.

Blood was collected in a volume of 10 mL in specialized containers containing anticoagulant K2EDTA. Within 15 min of blood collection, samples were centrifuged at room temperature for 15 min at 3,000 rpm (1,600 × g) to separate plasma from cellular components. The resulting plasma was transferred into a specialized plastic container and tightly sealed, after verifying the patient’s name and other identifying information on the registration form. The container was labeled with the patient’s information. The separated plasma was transferred into labeled polypropylene tubes, aliquoted, and stored at −80 °C until analysis. It was ensured that repeated freeze–thaw cycles were avoided. All samples were processed according to a standardized pre-analytical protocol to minimize analytical variability. The same sample collection, centrifugation, aliquoting, storage, and handling procedures were applied to all participants before metabolomic analysis. The analytical procedure was performed using a proprietary HPLC-MS method for quantitative determination of amino acids in EDTA plasma, routinely implemented in the study laboratory according to standardized operating procedures. Quality assurance procedures were implemented throughout sample preparation and instrumental analysis to ensure analytical reproducibility.

The laboratory portion of the study was carried out in the scientific research laboratory of NJSC Karaganda Medical University. Plasma concentrations of six amino acids were determined using high-performance liquid chromatography coupled with mass spectrometry (HPLC-MS).

### 2.5 The analytical platform of HPLC-MS

Quantitative determination of plasma amino acids was performed using an Agilent 1260 Infinity HPLC system coupled to an Agilent 6130B single-quadrupole mass-selective detector (Agilent Technologies, USA). Chromatographic separation was performed on a Zorbax Eclipse Plus Rapid Resolution C18 analytical column (4.6 × 100 mm, 3.5 μm) protected by a Zorbax Eclipse Plus C18 guard column (4.6 × 12.5 mm, 5 μm). The column temperature was maintained at 35 °C.

Plasma samples were prepared using protein precipitation. Briefly, 100 μL of plasma was mixed with 600 μL of acetonitrile, vortex-mixed for 30 s, centrifuged at 12,000 × g for 10 min at 4 °C, and 100 μL of the resulting supernatant was transferred to an autosampler vial and diluted with an equal volume of deionized water before analysis.

Mobile phase A consisted of 0.2% formic acid in water, whereas mobile phase B consisted of 0.2% formic acid in acetonitrile. Gradient elution was performed as follows: 1% B (0–1 min), 60% B (1–7 min), 90% B (7.1–8.4 min), and 1% B (8.6–13.0 min).

The flow rate was 0.5 mL/min, the injection volume was 10 μL, and the total run time was 13 min. Detection was performed using electrospray ionization in positive-ion mode with selected-ion monitoring (SIM) of [M + H] + ions. Amino acids were identified by comparison of retention times with reference standards. Quantification was performed using the external standard method. Isotopically labelled internal standards were not used.

### 2.6 Quality assurance and data management

Analytical quality control included the analysis of blank samples, control samples, and repeated injections of selected samples throughout the analytical sequence. Retention-time stability, chromatographic peak shape, baseline stability, and signal reproducibility were monitored during analysis. Data processing was performed using Agilent software with uniform peak-integration settings applied to all analytical runs. Only chromatographic peaks meeting predefined quality criteria were included in the analysis, whereas samples exhibiting analytical artifacts were excluded.

### 2.7 Statistical analysis

Statistical analyses were performed using Statistica software (StatSoft Inc., Tulsa, OK, USA), except for receiver operating characteristic (ROC) curve analysis, which was performed using Jamovi version 2.7.18. Analyses involving routine clinical and laboratory variables were performed using an available-case approach; no imputation of missing values was performed. Consequently, each statistical analysis included all participants with complete data for the variables under investigation. The normality of continuous variables was assessed using the Shapiro–Wilk and Kolmogorov–Smirnov tests with Lilliefors correction. As most continuous variables were not normally distributed, data are presented as the median and interquartile range (IQR; Q1–Q3). All statistical tests were two-sided, and a *p-*value <0.05 was considered statistically significant.

Differences in continuous variables among study groups were assessed using the Kruskal–Wallis test. When the overall test was statistically significant, pairwise comparisons were performed using Dunn’s *post-hoc* test with adjustment for multiple comparisons, and adjusted *p-*values were reported. Within-group changes in amino acid concentrations between the two sampling time points were evaluated using the Wilcoxon signed-rank test based on *Δ*-values (difference between measurements obtained at 12 h and 3–4.5 h after symptom onset). Correlations between metabolite concentrations and clinical, laboratory, and instrumental variables, including NIHSS scores, were assessed using Spearman’s rank correlation coefficient.

The diagnostic performance of individual metabolites and multivariable models was evaluated using ROC curve analysis by calculating the area under the curve (AUC), sensitivity, and specificity. Multivariable logistic regression analysis was performed to identify independent predictors of acute ischemic stroke and to evaluate the incremental diagnostic value of metabolomic markers in combination with clinical variables. All multivariable logistic regression models were developed using all participants with complete data for the variables included in each model. Variables demonstrating clinical relevance and/or statistical significance in univariable analyses were considered for inclusion in the multivariable models. Odds ratios (ORs) with 95% confidence intervals (95% CIs) were reported as measures of effect size for the multivariable logistic regression models. Differences between ROC curves were assessed using the DeLong test.

## 3 Results

### 3.1 Basic characteristics of the study population

The current study involved 297 participants, comprising 122 individuals with AIS, 50 with HS, 50 with TIA, and 75 individuals of the control group. These groups were similar in terms of gender and age, as detailed demographic and clinical information is presented in Table 1. There was a difference in median age between the groups, which was statistically significant (*p* = 0.005). However, only a statistically significant difference was found between the AIS group and the control group, with respect to this variable (*p* = 0.004).

**Table 1:** Baseline clinical and demographic characteristics of patients in the study groups.

Indicator	All patients (*n* = 297)	AIS (*n* = 122)	HS (*n* = 50)	TIA (*n* = 50)	Control group (*n* = 75)
Age, years, Ме (IQR)	65.0 [55.0–74.0]	67.5 [60.0–75.0]	63.5 [53.0–72.0]	67.5 [54.0–74.0]	60.0 [47.0–71.0]
Male sex, *n* (%)	170 (57.2)	72 (59.0)	33 (66.0)	32 (64.0)	33 (44.0)
BMI, Ме (IQR), kg/m^2^	27.69 [24.22–31.60]	28.53 [25.27–32.34]	28.41 [23.88–33.20]	26.24 [23.45–29.22]	26.18 [24.17–31.22]
Event index**, Me [IQR] min	120 [80–160]	120 [80–160]	90 [70–125]	120 [90–180]	NA
Stroke onset time***, *n* (%)					
Night (00:00–05:59)	14 (6.3)	8 (6.6)	5 (10.0)	1 (2.0)	NA
Morning (06:00–11:59)	91 (41.0)	50 (41.0)	16 (32.0)	25 (50.0)	NA
The day (12:00–17:59)	78 (35.1)	42 (34.4)	18 (36.0)	18 (36.0)	NA
Evening (18:00–23:59)	39 (17.6)	22 (18.0)	11 (22.0)	6 (12.0)	NA
Concomitant endocrine disorders					
Absent, *n* (%)	206 (69.4)	84 (68.9)	34 (68.0)	30 (60.0)	58 (77.4)
Diabetes mellitus, *n* (%)	37 (12.4)	11 (9.0)	10 (20.0)	12 (24.0)	4 (5.3)
Thyroid diseases, *n* (%)	14 (4.7)	2 (1.6)	3 (6.0)	6 (12.0)	3 (4.0)
Obesity/metabolic syndrome, *n* (%)	17 (5.7)	9 (7.4)	3 (6.0)	2 (4.0)	3 (4.0)
Impaired glucose tolerance, *n* (%)	5 (1.7)	1 (0.8)	0	0	4 (5.3)
Multiple diseases, *n* (%)	18 (6.1)	15 (12.3)	0	0	3 (4.0)

**Event index denotes the time from symptom onset to hospital admission, expressed in minutes. ***Stroke onset time denotes the clock time at which acute neurological symptoms began; this variable is not applicable to control participants.

The data are presented as median and interquartile range (Me [IQR]) and absolute and relative values [*n* (%)]. Percentage values for categorical variables were calculated from valid observations. “Other” category includes nationalities with a small number of observations (<5 in each subgroup). Data on nationality was missing for 8 (2.7%) patients. In the control group, the event index could not be calculated due to lack of an acute vascular event. The control group was excluded from the analysis as the time of stroke onset was not determined for this group. Body mass index (BMI) was calculated for 198 patients. AIS, acute ischemic stroke; HS, hemorrhagic stroke; TIA, transient ischemic attack; BMI, body mass index.

The overall sample was predominantly male (57.2%). Intergroup differences in gender were also statistically significant (*p* = 0.045), with a small effect size. Additionally, women exhibited a higher severity of stroke as measured by the NIHSS scale (*p* = 0.007), and there was a weak positive correlation between age and NIHSS (*r* = 0.22, *p* < 0.05).

There was no significant difference in the median time from symptom onset to hospitalization between clinical groups (120 min [80–160]), and there were no differences in BMI or circadian patterns of stroke onset. The most prevalent comorbidities in the study population were cardiovascular conditions, specifically arterial hypertension. Other clinical variables did not differ significantly between groups and were not found to have a significant impact on the outcomes of the analysis.

The clinical characteristics of the study participants are presented in Table 2. Among patients with AIS, the middle cerebral artery basin was most commonly affected, predominantly on the left side. In contrast, the localization pattern in patients with TIA was more evenly distributed. In these individuals, damage to the vertebrobasilar artery basin predominated.

**Table 2:** Clinical characteristics, vascular pools, and pathogenetic subtypes of stroke in the study groups.

Indicator	All patients (*n* = 297)	AIS (*n* = 122)	HS (*n* = 50)	TIA (*n* = 50)	Control group (*n* = 75)
Vascular territory					
Left middle cerebral artery *n* (%)	73 (32.9)	53 (43.4)	15 (30.0)	5 (10.0)	NA
Right middle cerebral artery, *n* (%)	76 (34.2)	44 (36.1)	20 (40.0)	12 (24.0)	NA
Right anterior cerebral artery, *n* (%)	1 (0.5)	1 (0.8)	0	0	NA
Vertebro-basilar basin, *n* (%)	64 (28.8)	20 (16.4)	11 (22.0)	33 (66.0)	NA
Right internal carotid artery, *n* (%)	4 (1.8)	1 (0.8)	3 (6.0)	0	NA
Several vascular basins*, *n* (%)	2 (0.9)	2 (1.6)	0	0	NA
AIS etiological subtypes (TOAST classification)					
Atherothrombotic *n* (%)	NA	79 (64.8)	NA	NA	NA
Cardioembolic, *n* (%)	NA	17 (13.9)	NA	NA	NA
Unknown etiology, *n* (%)	NA	20 (16.4)	NA	NA	NA
Other established etiologies, *n* (%)	NA	6 (4.9)	NA	NA	NA
Stroke severity and functional status assessment scales, Me [IQR]					
NIHSS score, Ме (IQR)	7.5 [4.0–12.0] (*n* = 222)	8.0 [5.0–12.0] (*n* = 122)	10.0 [7.0–15.0] (*n* = 50)	3.0 [3.0–6.0] (*n* = 50)	NA
GCS score, Ме (IQR)	15 [14–15] (*n* = 222)	15 [14–15] (*n* = 122)	14 [12–15] (*n* = 50)	15 [15–15] (*n* = 50)	NA
MMSE score, Ме (IQR)	23.0 [18.0–27.0] (*n* = 133)	19.0 [17.0–21.0] (*n* = 55)	18.5 [10.0–20.0] (*n* = 18)	28.0 [27.0–30.0] (*n* = 26)	28.0 [26.0–29.0] (*n* = 34)
The degree of arterial hypertension upon admission					
0, *n* (%)	3 (1.0)	3 (2.5)	0	0	0
I, *n* (%)	8 (2.7)	7 (5.7)	1 (2.0)	0	0
II, *n* (%)	18 (6.1)	16 (13.1)	2 (4.0)	0	0
III, *n* (%)	268 (90.2)	96 (78.7)	47 (94.0)	50 (100.0)	75 (100.0)

*“Several vascular basins” denotes multifocal involvement of more than one cerebral vascular territory as determined by neuroimaging.

The data presented are in the form of median and interquartile range (Me [IQR]) for quantitative variables, and absolute and relative values [*n* (%)] for categorical variables. Percentages are calculated based on the number of valid observations. Pathogenetic subtypes of stroke are only provided for the ischemic stroke group, and the designation “not applicable” has been used for indicators that were not evaluated in the respective groups. The category “multiple vascular pools” represents multifocal damage as determined by neuroimaging data.

The atherothrombotic subtype of AIS was the most prevalent (64.8%), with the cardioembolic subtype and stroke of unknown etiology being significantly less common. However, there were no significant differences in amino acid levels between the different subtypes of stroke (*p* > 0.05).

The severity of neurological impairments on the NIHSS scale differed significantly between the groups (*p* < 0.001). The most pronounced deficits were observed in patients with HS and AIS, while in TIA, the scores were significantly lower. No significant difference was found between AIS and HS groups according to the NIHSS score (*p* = 0.397).

The level of consciousness as measured by the Glasgow Coma Scale (GCS) also differed significantly among the groups (*p* < 0.001), with the greatest decrease observed in the HS group. Cognitive function as assessed by the Mini-Mental State Examination (MMSE) scale was significantly lower in patients with stroke compared to those with TIA and the control group (*p* < 0.001), with a greater reduction in individuals with HS.

Arterial hypertension is prevalent in all groups of patients, primarily in grade III, reflecting the high cardiovascular morbidity of the examined population. The neurological deficit corresponds to the clinical presentation of stroke, with greater motor and speech deficits in patients with AIS and HS compared to TIA. Elevated blood pressure levels at the time of vascular event were observed in all groups, with the highest values in patients with pre-existing hypertension. A reduction in systolic blood pressure was noted over time, although significant variability persisted in patients with HS. An increase in body temperature was more frequently observed in patients with HS compared to AIS.

### 3.2 Neuroimaging features of the patients under study

The neuroimaging findings of the patients are presented in Table 3. At admission, signs of acute stroke based on CT/MRI scans were identified in 41.8% of the overall cohort, indicating the limited sensitivity of this method in the early stages of the condition. In the HS group, focal abnormalities were visualized in nearly all cases, while in the AIS group, they were detected in fewer than half of the patients. No focal changes were observed in patients with TIA at admission.

**Table 3:** Clinical and neuroimaging characteristics of the study groups.

Indicator	All patients with acute cerebrovascular disease (*n* = 222)	AIS (*n* = 122)	HS (*n* = 50)	TIA (*n* = 50)
Signs of stroke on admission to the stroke center based on CT/MRI scan, *n* (%)				
Absent	126 (56.8)	76 (62.3)	0	50 (100.0)
Present	93 (41.8)	45 (36.9)	48 (96.0)	0
Not conducted	3 (1.4)	1 (0.8)	2 (4.0)	0
Signs of stroke on CT/MRI within 24 h of admission to stroke center**, *n* (%)				
Absent	62 (34.6)	35 (32.1)	0	27 (100.0)
Present	117 (65.4)	74 (67.9)	43 (100.0)	0
No data available	43	13	7	23
Intracerebral hematoma volume at admission, mL, Ме (IQR)	NA	NA	12.1 [5.2–28.2] (*n* = 43)	NA
Hemorrhagic transformation/intracerebral hematoma within 24 h (*n*, %)				
Absent	100 (58.1)	96 (78.7)	4 (8.0)	—
Present	48 (27.9)	9 (7.4)	39 (78.0)	—
No data available	24 (14.0)	17 (13.9)	7 (14.0)	—
SAH/IVH according to CT/MRI data 24 h after admission to the IC, *n* (%)				
Absent	143 (83.1)	110 (90.2)	33 (66.0)	—
SAH	4 (2.3)	0	4 (8.0)	—
IVH	6 (3.5)	0	6 (12.0)	—
No data available	19 (11.1)	12 (9.8)	7 (14.0)	—

The data presented are given in absolute values and as percentages. The percentage values have been calculated based on the total number of valid observations within each group. Due to the absence of acute cerebrovascular events, the control group has not been included in the analysis. **The percentages have been calculated using the number of patients who underwent CT/MRI 24 h after admission; the “No Data Available” line represents the absolute number of patients without a control neuroimaging study. SAH, subarachnoid hemorrhage; IVH, intraventricular hemorrhage.

Following control neuroimaging, stroke signs were seen in most patients after 24 h, whereas in the HS group, abnormalities persisted in all patients.

Hemorrhagic conversion of AIS was rare, while signs of intracranial, subarachnoid, and intraventricular bleeding were primarily recorded in patients with high-intensity lesions.

The data obtained support the limited diagnostic accuracy of neuroimaging during the early stages of AIS and underscore the need to explore additional biomarkers for an early differential diagnosis.

### 3.3 Laboratory characteristics of the patients under study

Laboratory parameters at admission indicated activation of systemic inflammatory and metabolic responses (Table 4). Among patients with acute cerebrovascular disease, there was an increase in leukocyte count compared to the control group (*p* < 0.001), as well as significant alterations in the leukocyte differential, including a reduction in the proportion of lymphocytes and an elevation of neutrophils, accompanied by an increase in the neutrophil-to-lymphocyte ratio (NLR) (*p* < 0.001). These changes were most pronounced in the TIA group. The erythrocyte sedimentation rate (ESR) was statistically significantly elevated in patients with AIS compared to other groups (*p* < 0.001).

**Table 4:** Laboratory parameters at admission in patients with acute cerebrovascular disorders.

Parameter	All patients	AIS	HS	TIA	Control group	*р*
Blood test						
Platelets, ×10^9^/L	222.6 [192.0–272.0] (*n* = 297)	217.5 [178.0–257.0] (*n* = 122)	223.6 [194.8–302.0] (*n* = 50)	245.0 [213.0–274.0] (*n* = 50)	222.2 [192.0–289.4] (*n* = 75)	0.035
Lymphocytes, %	23.1 [18.4–33.4] (*n* = 80)	33.5 [16.0–39.9] (*n* = 9)	31.4 [8.4–33.4] (*n* = 7)	10.8 [7.7–12.2] (*n* = 14)	26.5 [21.6–33.6] (*n* = 50)	<0.001
Neutrophils, %	68.5 [59.0–84.4] (*n* = 80)	71.3 [58.0–82.2] (*n* = 9)	60.7 [57.3–83.3] (*n* = 7)	86.0 [82.2–86.4] (*n* = 14)	67.0 [59.0–72.0] (*n* = 50)	0.007
NLR	2.6 [1.8–4.3] (*n* = 76)	3.0 [1.5–5.9] (*n* = 9)	1.9 [1.7–10.0] (*n* = 7)	7.9 [7.1–11.2] (*n* = 14)	2.1 [1.7–3.0] (*n* = 46)	<0.001
White blood cells, ×10^9^/L	7.4 [6.2–9.5] (*n* = 293)	7.6 [6.7–9.6] (*n* = 122)	8.1 [7.0–11.7] (*n* = 50)	8.3 [6.6–10.5] (*n* = 50)	6.3 [5.2–7.6] (*n* = 71)	<0.001
ESR, mm/h	9.0 [5.0–14.0] (*n* = 286)	11.0 [7.0–16.0] (*n* = 121)	8.0 [5.0–14.0] (*n* = 49)	6.0 [3.0–13.0] (*n* = 43)	5.0 [4.0–9.0] (*n* = 73)	<0.001
Coagulogram						
APTT, s	31.0 [28.8–33.6] (*n* = 265)	32.0 [30.0–35.0] (*n* = 122)	31.0 [29.0–34.0] (*n* = 50)	30.4 [26.8–32.5] (*n* = 50)	28.9 [26.0–31.9] (*n* = 43)	<0.001
International normalized ratio	1.0 [1.0–1.1] (*n* = 266)	1.1 [1.0–1.1] (*n* = 122)	1.1 [1.0–1.1] (*n* = 50)	1.0 [0.9–1.0] (*n* = 50)	1.0 [0.9–1.1] (*n* = 44)	0.072
PT, s	15.0 [11.1–17.0] (*n* = 263)	16.0 [15.0–17.0] (*n* = 122)	15.0 [11.2–17.0] (*n* = 50)	11.8 [10.9–15.0] (*n* = 50)	11.1 [10.6–12.3] (*n* = 41)	<0.001
PI, %	97.2 [90.9–104.1] (*n* = 266)	94.0 [94.0–100.0] (*n* = 122)	94.0 [88.2–105.0] (*n* = 50)	102.3 [94.0–105.0] (*n* = 50)	99.7 [89.6–105.5] (*n* = 44)	0.080
Fibrinogen, g/L	3.7 [3.4–4.2] (*n* = 266)	3.7 [3.4–3.9] (*n* = 122)	3.7 [3.4–3.9] (*n* = 50)	4.0 [2.8–4.7] (*n* = 50)	3.7 [2.9–5.5] (*n* = 44)	0.214
Biochemical blood analysis						
Glucose, mmol/L	6.5 [5.7–7.6] (*n* = 261)	6.8 [5.8–8.0] (*n* = 119)	6.5 [5.9–7.6] (*n* = 49)	6.8 [5.8–7.5] (*n* = 47)	5.7 [4.9–6.4] (*n* = 46)	<0.001
ALT, IU/L	20.6 [15.6–30.6] (*n* = 268)	21.2 [16.2–29.8] (*n* = 122)	17.3 [13.5–21.0] (*n* = 50)	23.2 [15.9–41.0] (*n* = 50)	20.5 [14.5–31.4] (*n* = 46)	0.003
AST, IU/L	23.0 [18.2–29.2] (*n* = 268)	23.8 [18.4–30.5] (*n* = 122)	22.6 [19.2–25.8] (*n* = 50)	22.0 [18.6–31.4] (*n* = 50)	20.0 [16.4–27.7] (*n* = 46)	0.375
Total protein, g/L	70.3 [66.3–74.1] (*n* = 256)	68.9 [65.0–72.6] (*n* = 118)	69.0 [65.6–74.0] (*n* = 49)	71.6 [67.0–77.0] (*n* = 47)	71.9 [68.4–76.1] (*n* = 42)	0.004
Total cholesterol, mmol/L	4.7 [4.0–5.9] (*n* = 259)	4.4 [3.7–5.0] (*n* = 118)	4.5 [3.4–5.6] (*n* = 49)	5.3 [4.6–6.4] (*n* = 48)	7.0 [5.0–8.3] (*n* = 44)	<0.001
LDL, mmol/L	2.6 [2.1–3.5] (*n* = 128)	2.7 [2.3–3.2] (*n* = 29)	2.3 [1.9–2.6] (*n* = 25)	2.9 [2.1–3.6] (*n* = 30)	3.0 [2.3–3.8] (*n* = 44)	0.190
HDL, mmol/L	1.4 [1.2–1.8] (*n* = 127)	1.5 [1.1–1.9] (*n* = 29)	1.4 [1.3–1.6] (*n* = 25)	1.3 [1.2–1.7] (*n* = 30)	1.4 [1.2–2.2] (*n* = 43)	0.821
Triglycerides, mmol/L	1.4 [0.9–2.0] (*n* = 132)	1.3 [0.8–2.0] (*n* = 31)	1.1 [0.9–2.0] (*n* = 27)	1.8 [1.2–2.7] (*n* = 30)	1.3 [0.9–1.8] (*n* = 44)	0.009
K, mmol/L	3.9 [3.6–4.1] (*n* = 186)	3.7 [3.4–4.0] (*n* = 62)	3.9 [3.6–4.0] (*n* = 39)	3.8 [3.6–4.1] (*n* = 41)	4.0 [3.9–4.1] (*n* = 44)	0.002
Ca, mmol/L	2.3 [1.0–2.3] (*n* = 135)	1.1 [0.7–2.3] (*n* = 45)	2.3 [1.0–2.4] (*n* = 31)	2.2 [1.6–2.4] (*n* = 31)	2.3 [2.3–2.3] (*n* = 28)	0.006
Cl, mmol/L	102.0 [101.0–105.0] (*n* = 126)	103.9 [102.0–106.0] (*n* = 41)	102.0 [100.0–104.0] (*n* = 30)	102.0 [100.0–106.0] (*n* = 27)	102.0 [101.0–102.0] (*n* = 28)	0.013
Na, mmol/L	139.4 [137.0–141.0] (*n* = 186)	141.0 [138.5–144.0] (*n* = 62)	138.9 [137.0–141.0] (*n* = 39)	138.0 [136.7–142.0] (*n* = 41)	139.0 [138.0–140.5] (*n* = 44)	0.008
Urea, mmol/L	6.3 [5.0–7.9] (*n* = 267)	6.7 [5.7–7.9] (*n* = 121)	6.2 [5.1–8.1] (*n* = 50)	6.9 [5.4–8.2] (*n* = 50)	4.9 [3.9–6.5] (*n* = 46)	<0.001
Creatinine, μmol/L	90.8 [74.7–110.0] (*n* = 266)	98.4 [82.3–111.6] (*n* = 120)	91.2 [76.3–123.7] (*n* = 50)	90.5 [74.2–110.0] (*n* = 50)	74.7 [66.0–94.0] (*n* = 46)	<0.001

Quantitative data are presented as median and interquartile range (Me [IQR]), while qualitative data are presented in absolute and relative terms (*n*, %). Differences between groups were assessed using the Kruskal–Wallis test.

Metabolic changes included an increase in blood glucose levels in patients with acute cerebrovascular events compared to the control group (*p* < 0.001). Additionally, there was an increase in levels of urea and creatinine, which may indicate a systemic stress response.

The analysis of lipid profiles revealed a decrease in total cholesterol and beta-lipoproteins among stroke patients compared to the control group (*p* < 0.001), along with differences in triglyceride levels between clinical groups. Coagulation indices (activated partial thromboplastin time and prothrombin time) showed moderate differences between groups, remaining within physiological ranges.

Overall, the observed changes were non-specific and reflected a systemic inflammatory and metabolic response to acute cerebrovascular events. These findings should be interpreted with caution because the availability of several routine laboratory parameters varied substantially among participants owing to retrospective data collection. Consequently, these analyses should be regarded as exploratory rather than definitive.

### 3.4 The amino acid profile in the initial stages of acute cerebrovascular disorders

**Figure 1:**
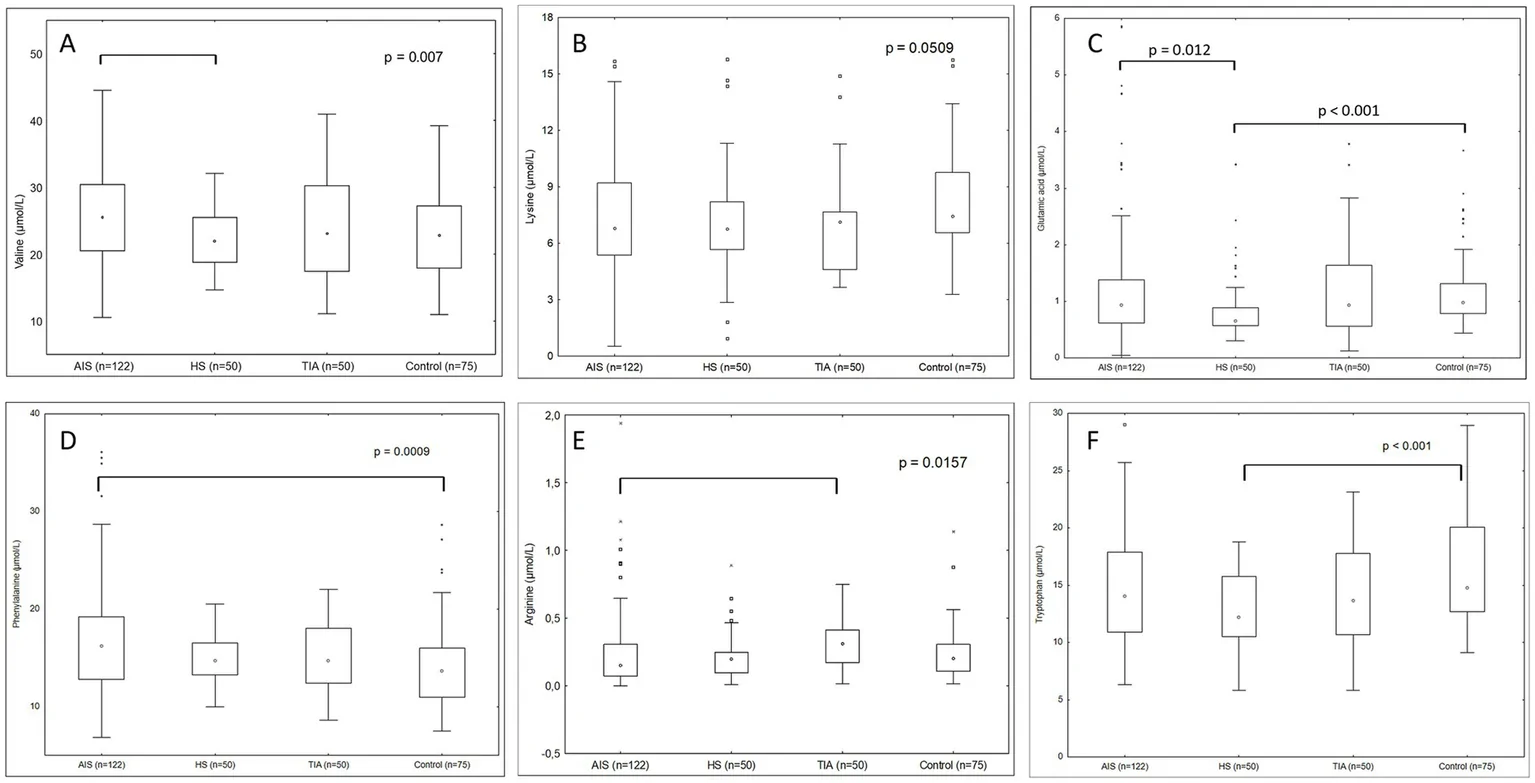
Distribution of plasma amino acid concentrations among patients with acute cerebrovascular events during the hyperacute phase. Box plots show the median (horizontal line within the box), interquartile range (box), and whiskers representing the non-outlier range (1.5 × IQR). Open circles indicate mean values, and individual dots represent outliers. Overall *p-*values were calculated using the Kruskal–Wallis test. Horizontal brackets indicate statistically significant pairwise differences identified by Dunn’s *post-hoc* test for multiple comparisons. **(A)** Valine; **(B)** lysine; **(C)** glutamic acid; **(D)** phenylalanine; **(E)** arginine; **(F)** Tryptophan.

At the first time point (3–4.5 h after the onset of symptoms), there were statistically significant differences between groups in the concentrations of several amino acids (see Table 5 and Figure 1).

**Table 5:** Concentrations of amino acids in plasma within the first 4.5 hours following the onset of symptoms.

The metabolite	All patients (*n* = 297)	AIS (*n* = 122)	HS (*n* = 50)	TIA (*n* = 50)	Control group (*n* = 75)
Valine, μmol/L	23.32 [18.98–28.88]	25.51 [20.47–30.50]	21.95 [18.72–25.55]	23.13 [17.38–30.28]	22.80 [17.88–27.26]
Lysine, μmol/L	7.08 [5.66–8.84]	6.78 [5.36–9.21]	6.75 [5.66–8.20]	7.11 [4.60–7.65]	7.41 [6.56–9.76]
Glutamic acid, μmol/L	0.89 [0.65–1.31]	0.93 [0.62–1.38]	0.66 [0.57–0.88]	0.93 [0.56–1.64]	0.98 [0.78–1.31]
Phenylalanine, μmol/L	14.97 [12.28–17.77]	16.19 [12.80–19.17]	14.66 [13.25–16.49]	14.68 [12.37–18.00]	13.64 [10.95–16.01]
Arginine, μmol/L	0.19 [0.09–0.31]	0.15 [0.07–0.31]	0.20 [0.09–0.25]	0.31 [0.17–0.41]	0.20 [0.11–0.31]
Tryptophan, μmol/L	13.86 [10.97–17.77]	14.02 [10.89–17.88]	12.21 [10.48–15.76]	13.66 [10.66–17.77]	14.76 [12.67–20.09]

The data are presented as median and interquartile range (Me [IQR]). The concentrations of amino acids were measured in plasma within the first 4.5 h following the onset of clinical symptoms. The number of participants (*n*) in each group is indicated in column headers. Overall between-group differences were assessed using the Kruskal–Wallis test; significant pairwise differences were evaluated using Dunn’s post-hoc test with adjustment for multiple comparisons.

Valine levels were higher in patients with AIS compared to those with HS and control groups (*p* = 0.007). This indicates a difference in metabolic response based on the type of stroke. Glutamic acid levels were lower in patients with HS compared to other groups (*p* = 0.0009).

Phenylalanine levels were significantly higher in AIS patients compared to the control group (*p* = 0.0015). There were differences in arginine concentrations between groups (*p* = 0.0157), with higher levels in TIA patients compared with AIS. Tryptophan concentrations were lower in TIA patients than in the control group (*p* = 0.0022), while lysine levels did not differ significantly between the groups (*p* > 0.05).

In general, the observed changes reflect early metabolic alterations in acute cerebrovascular disorders and demonstrate variations in the amino acid profile based on the clinical presentation of the stroke.

### 3.5 Metabolic changes at the second time point (up to 12 h after the onset of symptoms)

**Figure 2:**
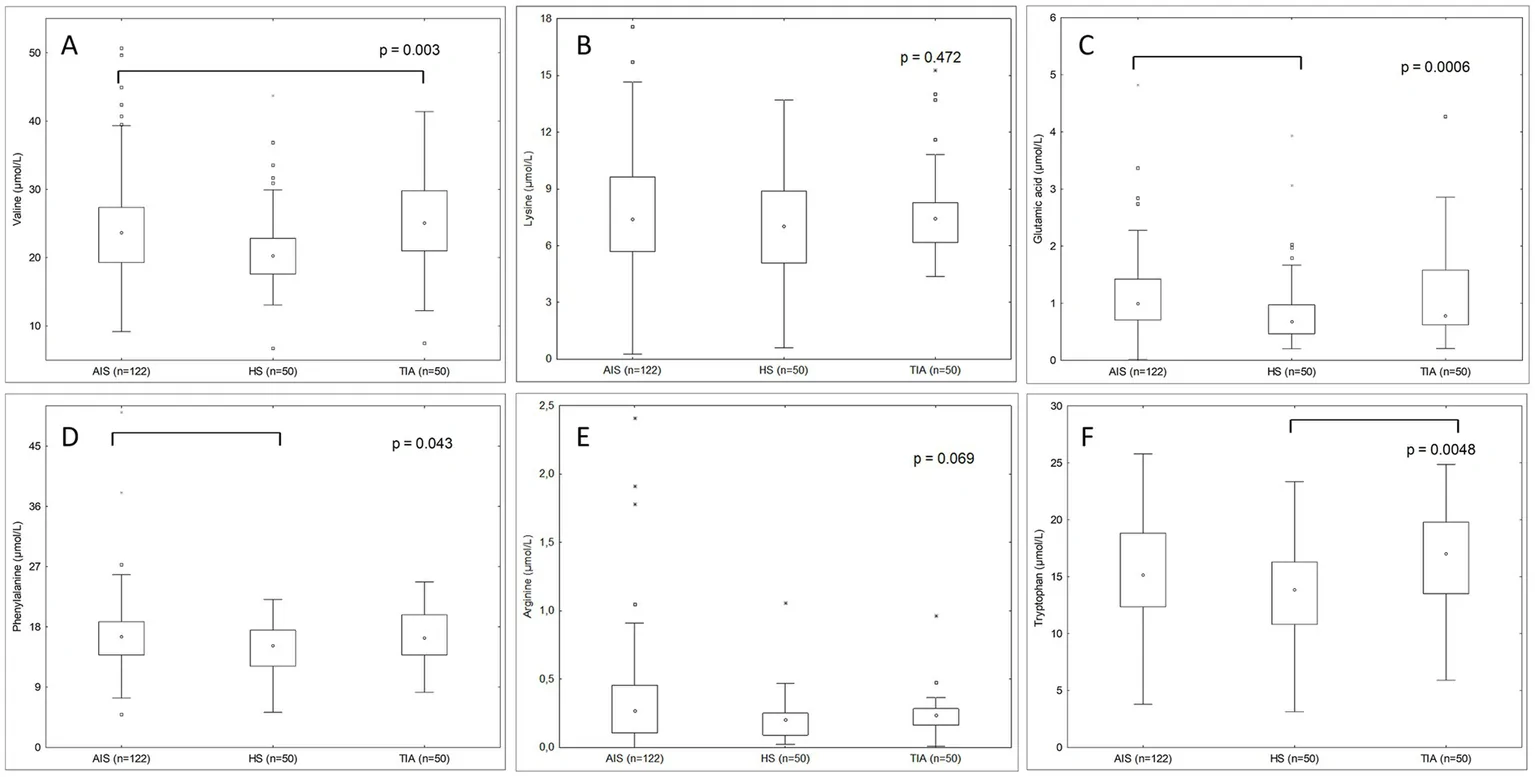
Distribution of plasma amino acid concentrations among patients with acute cerebrovascular events at the second time point (12 h after symptom onset). Box plots show the median (horizontal line within the box), interquartile range (box), and whiskers representing the non-outlier range (1.5 × IQR). Open circles indicate mean values, and individual dots represent outliers. Overall *p-*values were calculated using the Kruskal–Wallis test. Horizontal brackets indicate statistically significant *p*airwise differences identified by Dunn’s *post-hoc* multiple-comparison test. **(A)** Valine; **(B)** lysine; **(C)** glutamic acid; **(D)** phenylalanine; **(E)** arginine; **(F)** tryptophan.

At the second time point of the study, which was up to 12 h after the onset of neurological symptoms, statistically significant differences in the plasma concentrations of several amino acids remained between different clinical forms of acute cerebrovascular disease (Table 6 and Figure 2).

**Table 6:** Plasma amino acid concentrations in the first 12 h following the onset of symptoms.

The metabolite	All patients (*n* = 222)	AIS (*n* = 122)	HS (*n* = 50)	TIA (*n* = 50)
Valine, μmol/L	22.83 [19.19–27.47]	23.64 [19.30–27.36]	20.30 [17.60–22.83]	25.09 [20.99–29.81]
Lysine, μmol/L	7.38 [5.70–9.32]	7.38 [5.70–9.64]	7.02 [5.10–8.88]	7.42 [6.17–8.26]
Glutamic acid, μmol/L	0.86 [0.62–1.35]	0.99 [0.71–1.42]	0.68 [0.47–0.97]	0.77 [0.62–1.58]
Phenylalanine, μmol/L	15.96 [13.33–18.15]	16.57 [13.81–18.75]	15.17 [12.12–17.50]	16.33 [13.80–19.79]
Arginine, μmol/L	0.23 [0.12–0.37]	0.27 [0.11–0.46]	0.20 [0.09–0.25]	0.23 [0.16–0.28]
Tryptophan, μmol/L	15.02 [12.10–18.64]	15.15 [12.35–18.82]	13.83 [10.81–16.29]	17.03 [13.50–19.79]

The data are presented as median and interquartile range (Me [IQR]). The concentrations of amino acids were measured in plasma within the first 12 h following the onset of clinical symptoms. The number of participants (*n*) in each group is indicated in column headers. Overall between-group differences were assessed using the Kruskal–Wallis test; significant pairwise differences were evaluated using Dunn’s post-hoc test with adjustment for multiple comparisons.

Concentrations of valine differed significantly between the study groups (*p* = 0.003), with the lowest levels recorded in patients with HS and the highest levels in patients with TIA. *Post-hoc* analysis showed that valine levels were significantly lower in HS compared to AIS (*p* = 0.021) and TIA (*p* = 0.003). Lysine concentrations did not differ significantly between groups (*p* = 0.472). Glutamic acid concentrations also differed significantly among the groups (*p* = 0.0006), with maximum levels observed for AIS and minimum levels for HS. *Post-hoc* testing confirmed significant differences between these two groups (*p* = 0.0004). Statistically significant intergroup differences were observed for phenylalanine (*p* = 0.043), which was mainly due to a decrease in its concentration among patients with HS compared to AIS (*p* = 0.041). Arginine levels showed no statistically significant differences between study groups, although there was a trend towards intergroup variability (*p* = 0.069). The tryptophan concentration differed significantly between clinical groups (*p* = 0.0048), with the lowest values observed in patients with HS and the highest values in patients with TIA. Subsequent analysis revealed statistically significant differences between the HS and TIA groups (*p* = 0.0036).

Thus, at the second time point, valine, glutamic acid, phenylalanine, and tryptophan maintained the greatest diagnostic variability, whereas lysine and arginine exhibited no significant differences among the clinical forms of acute cerebrovascular disease.

### 3.6 The dynamics of amino acid concentrations within the first 12 h following the onset of a stroke

**Figure 3:**
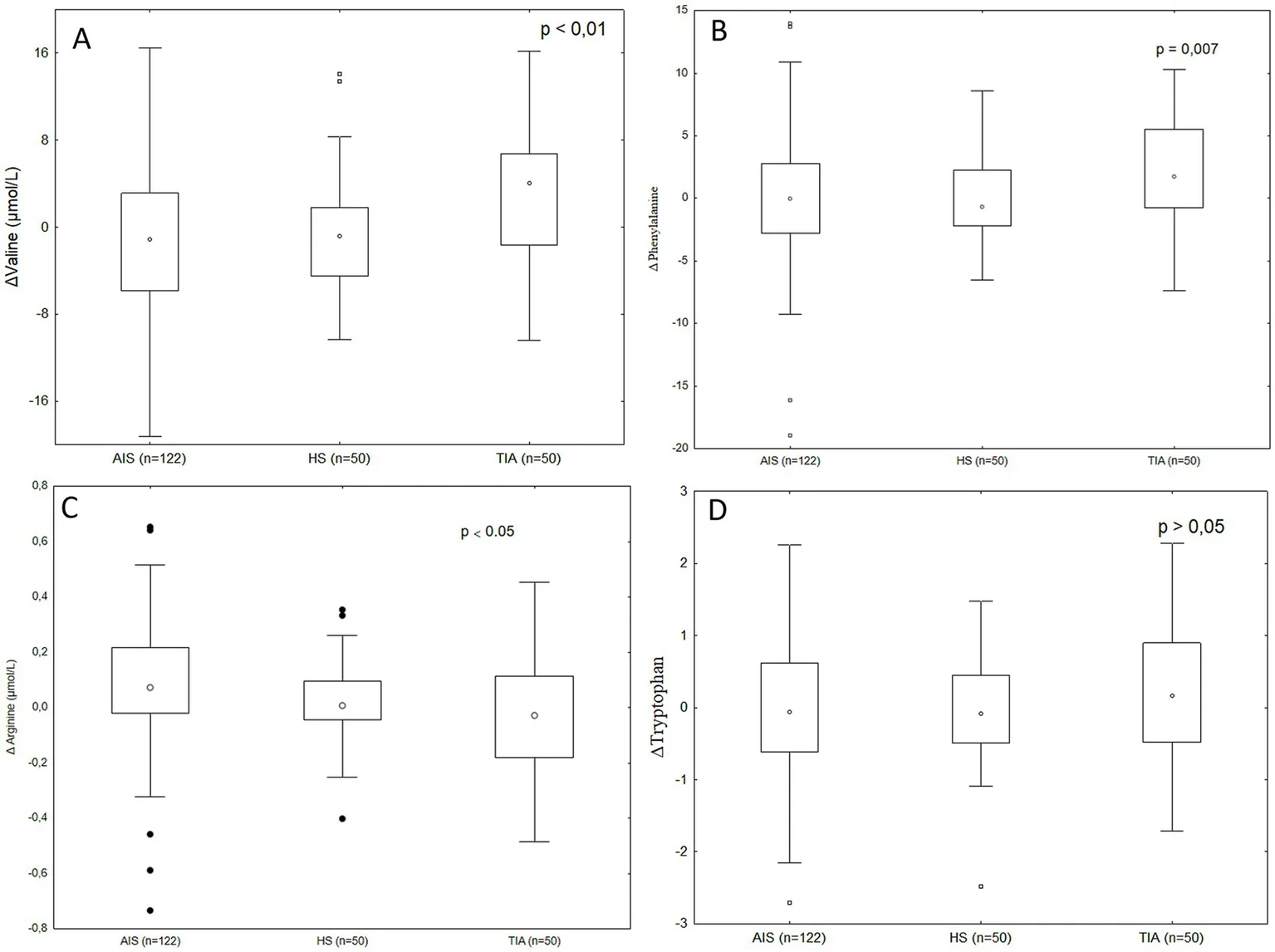
Changes in plasma amino acid concentrations between baseline and 12 h after symptom onset in patients with acute cerebrovascular diseases. Box plots show the median (horizontal line within the box), interquartile range (box), and whiskers representing the non-outlier range (1.5 × IQR). Open circles indicate mean values, and dots represent outliers. *Δ* values were calculated as the difference between the second and first sampling time points (12 h − baseline). Overall *p-*values were calculated using the Kruskal–Wallis test. **(A)** *Δ*Valine; **(B)** *Δ*phenylalanine; **(C)** *Δ*arginine; **(D)** *Δ*tryptophan.

Analysis of the changes in amino acid concentrations over time (*Δ* = t2 − t1) revealed a marked heterogeneity in metabolic changes depending on the clinical type of acute cerebrovascular disease (Figure 3). The most significant form-specific differences were observed for valine and phenylalanine.

In patients with AIS, there was a significant decrease in valine concentrations (*p* < 0.05). Conversely, in TIA, an increase in valine was noted (*p* = 0.019). A comparison between the two groups showed statistically significant differences in *Δ*-valine (*p* < 0.01), with higher concentrations in TIA patients compared to AIS and HS patients.

A similar trend was observed for phenylalanine, with a significant increase in concentrations only in TIA (*p* = 0.001). There were no significant changes in either AIS or HS groups. Intergroup differences in ∆phenylalanine also reached statistical significance (*p* < 0.05). Arginine dynamics were characterized by a statistically significant increase only in patients with AIS (*p* < 0.05), while intergroup differences in the ∆-index were also significant (*p* < 0.05), with higher values in the AIS group.

Unlike these amino acids, changes in lysine and tryptophan were less specific. An increase in lysine concentration was observed in AIS and TIA (*p* < 0.05), but intergroup differences in ∆-lysine did not reach statistical significance. Tryptophan showed a statistically significant increase in all study groups (*p* < 0.05), indicating the universal nature of metabolic dynamics regardless of the type of disease. Glutamic acid did not show significant changes in any group, and there were no differences between groups in the *Δ*-index, indicating the stability of this metabolite during the study period.

Therefore, the analysis of the *Δ*-indices allows us to identify specific patterns of metabolic changes, which may not be apparent when considering individual time points. Valine, phenylalanine, and arginine appear to be the most informative in this respect.

### 3.7 Diagnostic performance of selected amino acids in differentiating acute cerebrovascular disease subtypes

ROC curve analysis demonstrated that the extended clinico-metabolomic model showed better discrimination for AIS than the baseline clinical model. The baseline model included sex, age, and leukocyte count. In the initial adjusted multivariable model, age, sex, leukocyte count, glutamic acid, phenylalanine, and tryptophan were evaluated simultaneously. Leukocyte count, phenylalanine, and tryptophan remained independently associated with AIS, whereas age and glutamic acid did not retain statistical significance. Sex was also independently associated with AIS in the initial adjusted model (*p* = 0.048). Higher leukocyte count (OR = 1.24, 95% CI 1.06–1.45; *p* = 0.008) and phenylalanine concentration (OR = 1.34, 95% CI 1.19–1.52; *p* < 0.001) were associated with increased odds of AIS, whereas higher tryptophan concentration was associated with decreased odds of AIS (OR = 0.78, 95% CI 0.67–0.85; *p* < 0.001). Adjusted odds ratios for the laboratory and metabolomic predictors are presented in Table 7.

**Table 7:** Multivariable logistic regression model for differentiation of AIS and controls.

Predictor	Adjusted OR	95% CI	*p*
Leukocyte count	1.24	1.06–1.45	0.008
Phenylalanine	1.34	1.19–1.52	<0.001
Tryptophan	0.78	0.67–0.85	<0.001

OR, odds ratio; CI, confidence interval. ORs were obtained from the final multivariable logistic regression model after adjustment for the other variables included in the model.

**Figure 4:**
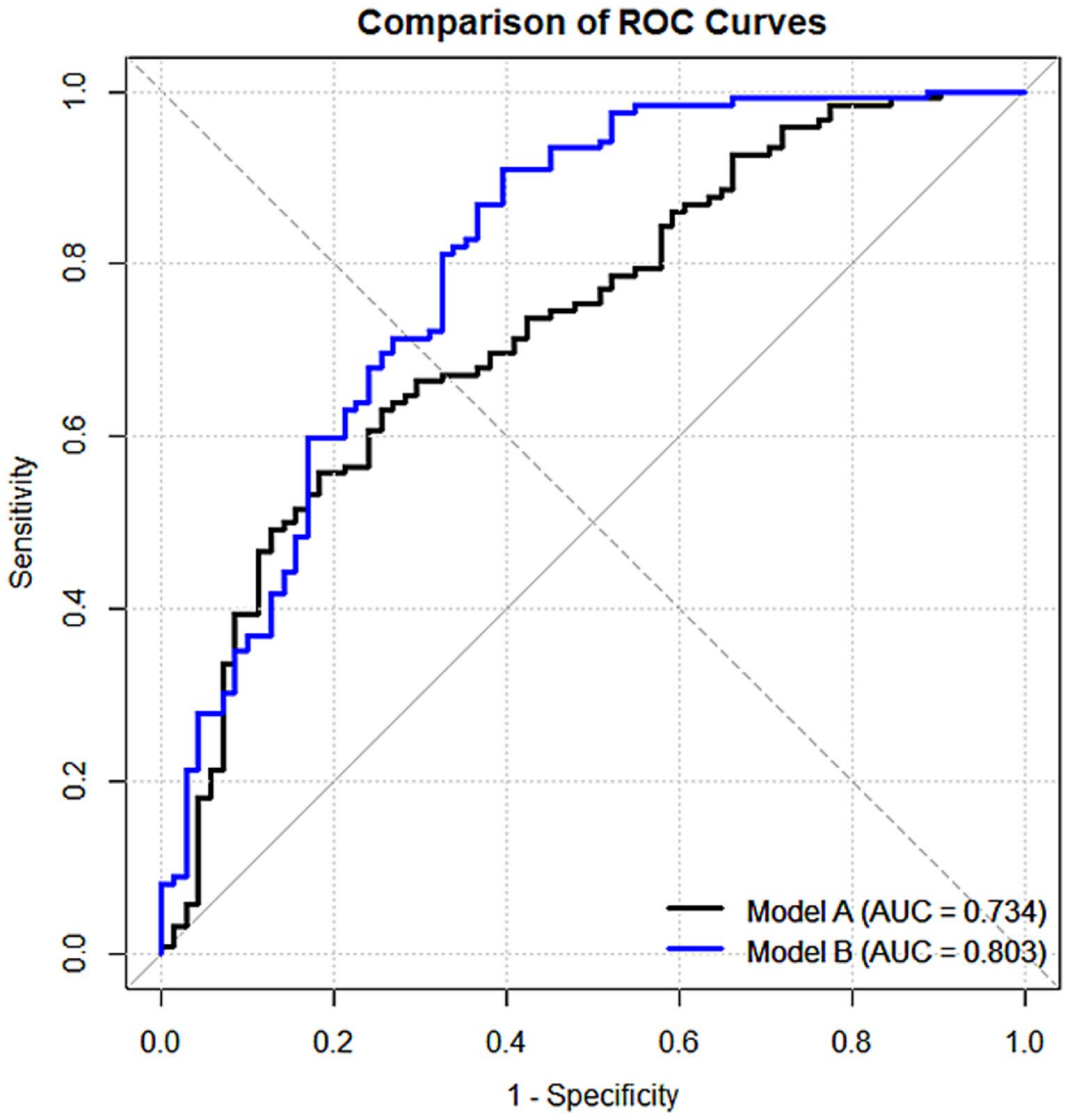
Receiver operating characteristic (ROC) curve analysis comparing the baseline clinical model and the extended clinico-metabolomic model for ischemic stroke diagnosis. The extended model (Model B, blue) showed superior discriminative ability compared to the baseline model (Model A, black), with an increase in AUC from 0.734 to 0.803. This improvement was statistically significant (DeLong test, *p* = 0.007).

ROC analysis of individual amino acids revealed limited discriminative ability (AUC range, 0.61–0.65). The baseline model had an AUC of 0.734, whereas the optimized clinico-metabolomic model achieved an AUC of 0.803. The difference between the models was statistically significant (DeLong test, *p* = 0.007), indicating an incremental improvement in discrimination after the addition of metabolomic variables within the development cohort (Figure 4).

## 4 Discussion

In this study, we conducted a comprehensive assessment of clinical, laboratory, and metabolic characteristics in patients with acute cerebrovascular events in order to identify diagnostic indicators in the hyperacute phase of stroke. A total of 297 participants were enrolled in the study (122 with AIS, 50 with HS, 50 with TIA, and 75 in the control group), allowing us to evaluate amino acid profiles based on clinical presentation.

### 4.1 Clinical and demographic characteristics

The analysis of clinical and demographic indicators in the patients has shown that the formed groups represent a typical structure of individuals with acute cerebrovascular disease. The distribution of the patients by gender, age, and main risk factors is generally in line with current epidemiological data.

Age is a significant risk factor for stroke, and it must be considered when interpreting metabolic alterations, as it may have a substantial impact on the concentrations of the amino acids under study. Therefore, in this study, patients with AIS were found to be statistically significantly older than those in the control group, in accordance with current epidemiological findings that indicate an increase in stroke incidence in older age groups (2, 11).

It should be noted that age can influence the metabolic profile of the body, through changes in energy metabolism, increased oxidative stress, and the development of chronic systemic inflammation. These changes are also accompanied by alterations in the amino acid profile (12). According to metabolic studies, age has been linked to changes in the concentrations of several amino acids and their metabolites, reflecting the specific features of protein and energy metabolism in older individuals (13, 14).

Another important demographic factor that may influence both the risk of stroke and its clinical course features is gender. In this study, among patients with acute cerebrovascular events in the overall sample, there was a male predominance (57.2%), which is consistent with epidemiological studies suggesting that stroke is more prevalent in men, particularly in younger age groups (15, 16).

One of the key factors in interpreting the metabolomic analysis is the time elapsed between the onset of acute neurological symptoms and hospitalization of patients with AIS. In this study, no statistically significant differences were observed between the clinical groups with regard to this indicator, suggesting that the groups are comparable and reducing the likelihood that a time factor influenced the observed differences in concentrations of the amino acids under study.

The next clinical and demographic factor that may influence the amino acid profile through its association with the development of insulin resistance, chronic systemic inflammation, and impaired energy metabolism, is body mass index (BMI) (17, 18). In this study, the indicator was found to be 27.69 kg/m^2^, which corresponds to the category of overweight. The data obtained is consistent with the findings of epidemiological research, which has shown a high prevalence of overweight and obesity in patients with cerebrovascular disease (19).

Despite the tendency towards a higher BMI among patients with AIS and HS conditions compared to those with TIA and the control group, no statistically significant differences were observed. This indicates that the studied groups are comparable in terms of BMI and reduces the likelihood that BMI influences the amino acid profile of the patients under study. Therefore, the observed changes in amino acid levels more accurately reflect the pathophysiological processes underlying stroke than the body weight characteristics of the participants.

The analysis of the patients’ background cardiovascular status revealed the presence of various types of cardiovascular disease in the majority of the individuals examined. The findings obtained are consistent with current epidemiological data, which indicate that hypertension is the most significant modifiable risk factor for stroke (19, 20).

Previous studies have demonstrated that long-term hypertension can lead to endothelial dysfunction, vascular remodeling, impaired microcirculation, and increased oxidative stress. These conditions are often accompanied by alterations in metabolic processes, such as amino acid metabolism (21, 22). In light of the above, it is important to consider the presence of hypertension among the study participants when interpreting the metabolic changes that were identified.

The localization of brain injury in stroke is a significant feature, as it provides insight into the characteristics of the vascular territory, the pathogenic mechanism of the condition, and the degree of damage to brain tissue (23). The findings demonstrate a typical pattern of lesion distribution in stroke. In AIS, the middle cerebral artery territory was most frequently affected, accounting for 43.4% of cases in the left hemisphere and 36.1% in the right, in line with global epidemiological data (24). In the HS group, the distribution of lesions across vascular territories was more uniform, with posterior cerebral artery involvement observed in 40% of cases. In contrast, TIA was characterized by a predominance of vertebrobasilar territory involvement (66%), likely reflecting pathogenetic differences between stroke subtypes with distinct lesion localization. Such a distribution may be explained by anatomical features of cerebral circulation and the large volume of brain tissue supplied by the middle cerebral artery. Given that lesion localization may be associated with the extent of ischemic injury and the severity of neurological deficit, this factor should be considered when interpreting the observed metabolic alterations.

The predominance of the atherothrombotic subtype of AIS (64.8%) is consistent with both global and regional epidemiological data based on the TOAST classification, in which the atherothrombotic subtype accounts for approximately 15–40% of cases. The proportions of the cardioembolic subtype (13.9%) and stroke of undetermined etiology (16.4%) are also in line with international registries, supporting the representativeness of the study groups for investigating the heterogeneity of acute cerebrovascular diseases (11, 24).

The median neurological deficit, assessed by the NIHSS, was 8.0 [5.0–12.0] in AIS and 10.0 [7.0–15.0] in HS, indicating moderate severity with a predominance of moderately severe cases (*p* < 0.001 for between-group differences). The level of consciousness, assessed by the Glasgow Coma Scale (GCS), was lower predominantly in HS [14 (12–15)], which may be related to the mass effect of intracerebral hemorrhage, whereas patients with TIA had preserved consciousness [15 (15)].

### 4.2 Neuroimaging findings and clinical correlations

The low detection rate of AIS during primary neuroimaging (36.9%) is consistent with the characteristics of the hyperacute phase of stroke (0–4.5 h), during which CT sensitivity to ischemic changes is limited and less than 50%, according to published data (5). It should be noted that, even when using MRI in the mode of diffusion-weighted imaging (DWI), an ischemic focus may not be visible, which has been observed in 6.9–23.2% of cases, according to the literature (25).

At the same time, the detection of bleeding in the HS group in all 100% of CT cases at admission and the lack of foci in TIA (0%) provided clear stratification of the study groups for subsequent analysis of metabolites. This emphasizes the limitations of neuroimaging techniques in the hyperacute phase of stroke and justifies the need for additional diagnostic markers, including metabolic parameters, to be sought.

A control neuroimaging performed 24 h later improved the verification of the diagnosis, with focal changes detected in most patients. The size of the intracranial hematoma in the HS (12.1 [5.2–28.2] mL), as well as the displacement of the midline structures, reflects the severity and extent of cerebral injury. The incidence of hemorrhagic transformation in AIS was low (7.4%), reducing the likelihood of it significantly affecting the analysis of metabolic alterations.

Neuroimaging data provide a basis for objective comparison with plasma metabolites studied, allowing us to evaluate their diagnostic value when used in conjunction with CT and MRI for early detection of stroke. It should be noted, however, that the variability in neuroimaging techniques used may affect the sensitivity in detecting focal changes, and consequently, the interpretation of results obtained.

### 4.3 Routine laboratory parameters and systemic response

Routine laboratory tests in patients with acute cerebrovascular disease reflect the complex systemic response of the body to acute cerebral injury, which includes changes in inflammation, metabolism, and hemostasis.

Changes in the leukocyte profile, such as an increase in neutrophils and a decrease in lymphocytes, as well as an increased NLR, are consistent with current understanding of the neuroinflammatory response in AIS. Neutrophils are the first immune cells to infiltrate ischemic tissue and contribute to blood–brain barrier disruption and secondary damage. Lymphopenia, on the other hand, may be a sign of post-stroke immunosuppression and the reallocation of immune resources in the body (26–28).

An increase in the NLR index, particularly in patients with TIA, may indicate an acute stress-induced response, accompanied by activation of the sympathetic-adrenal system and alterations in the leukocyte count, which can be regarded as a comprehensive indicator of neuroimmune activation. These changes are linked to the severity of the condition and an unfavorable prognosis (29, 30).

The laboratory findings support the concept of AIS as a systemic inflammation-metabolism condition, in which local brain tissue processes are accompanied by an intense peripheral immune response. According to current theories, the central and peripheral immune systems interact closely in a bidirectional manner, mediated by disruption of the blood–brain barrier, endothelial activation, and migration of immune cells to the ischemic area (27, 31, 32). This process is also accompanied by the development of systemic inflammation and post-stroke immune dysfunction, reflecting the complex nature of neuroimmune regulation in AIS (28).

Despite statistically significant differences in certain coagulation parameters (activated partial thromboplastin time and prothrombin time), their values generally remained within reference ranges, indicating the absence of marked hemostatic disturbances in the studied patients. The observed changes likely reflect reactive activation of the coagulation cascade in response to acute vascular injury and are consistent with current concepts of the thrombo-inflammatory nature of stroke (33).

However, these changes are not specific in nature and do not allow for a clear distinction between clinical groups. This indicates the limited usefulness of routine hemostatic indicators in interpreting metabolic changes identified in the study.

The biochemical analysis of blood samples from patients with acute cerebrovascular events revealed changes that reflect systemic metabolic stress associated with acute brain injury. Most notably, there was an increase in glucose levels. This is considered a key factor in secondary brain damage and contributes to increased oxidative stress, disruption of the blood–brain barrier, and the progression of cerebral infarction (34).

Additionally, the observed changes in protein and lipid metabolic parameters may indicate the activation of catabolic processes and a restructuring of energy metabolism under conditions of acute ischemia. It has been established that during the acute phase of stroke, there is a tendency towards hypercatabolism accompanied by the mobilization of energy reserves and a modification of the lipid profile. This is linked to the activation of neuroendocrine mechanisms and the body’s stress response (35).

In general, the identified biochemical changes are non-specific in nature and reflect universal mechanisms of the stress response and metabolic adaptation of the body. This limits their diagnostic value in differentiating between various forms of acute cerebrovascular disease. At the same time, these processes create a metabolic background that can influence the amino acid profile in blood.

### 4.4 Metabolomic profile at baseline

During the hyperacute phase of acute cerebrovascular events (within the first 4.5 h), patients experiencing AIS have been observed to experience an increase in valine levels, indicative of an early metabolic response to brain ischemia. Valine, one of the branched-chain amino acids, plays a role in energy metabolism and provides alternative substrates for cells under hypoxic conditions, where activation of catabolic processes and mobilization of the amino acid pool result in an elevation of its concentration in the systemic circulation (7, 8).

The data obtained is consistent with the findings of metabolic studies, which demonstrate a link between changes in the levels of branched-chain amino acids and impaired mitochondrial function. This is accompanied by activation of protein breakdown and systemic metabolic stress. Additionally, there may be an increase in valine levels during the acute phase, followed by a decline in later stages of stroke (6, 36).

Therefore, the observed increase in valine concentration may represent the hyperacute phase of the adaptive metabolic response related to energy deficiency and the stress response of the body.

Lysine, as a vital amino acid, plays a crucial role in protein and structural metabolic processes and is not a major component of rapid energy production or neurotransmission reactions. Within the first 4.5 h following the onset of ischemic stroke, there were no statistically significant differences between groups in its concentration, indicating a lack of pronounced changes in this metabolite during the hyperacute phase. This finding is consistent with previous research (37).

At the same time, metabolic data suggest the involvement of lysine in systemic metabolic reorganization due to the activation of its catabolic pathways (7). It is likely that the stability of the plasma level of lysine is due to a balance between mobilization and utilization, which prevents the development of significant fluctuations in concentration.

Therefore, lysine, unlike other metabolites directly involved in energy metabolism, does not serve as a sensitive marker for plasma levels in the hyperacute phase of stroke.

In the hyperacute phase of acute cerebrovascular disease, differences between groups were observed in terms of glutamic acid levels, primarily due to a decrease in these levels in patients with HS. Conversely, in patients with AIS, TIA, and the control group, the levels were similar. This suggests that metabolic differences may begin to form early, depending on the pathogenic variant of the condition.

Glutamic acid is an important component of amino acid metabolism and plays a role in regulating the glutamate-glutamine cycle. Under conditions of ischemia, when metabolic processes are reorganized, including energy deficiency and mitochondrial dysfunction, changes in plasma levels of glutamic acid may not be significant in the first few hours. This explains the similarity in levels between the different groups (7, 8).

At the same time, a reduction in glutamic acid concentration in cases of HS likely reflects a distinct type of injury associated with mechanical damage to tissue and the toxic effects of breakdown products of blood, which may be accompanied by a relative reduction in metabolic activity.

Considering the limited data available on plasma glutamic acid levels in patients with HS (37), the results obtained contribute to a better understanding of the metabolic characteristics of this type of cerebral vascular injury.

Therefore, a reduction in the level of glutamic acid during the hyperacute phase can be considered a characteristic feature of HS.

In the hyperacute phase of acute cerebrovascular events, patients with AIS demonstrated a statistically significant elevation in phenylalanine levels compared to the control group, suggesting an early activation of systemic metabolic responses. However, the lack of significant differences between other patient groups indicates that this indicator has limited discriminatory ability in the initial hours of illness.

Phenylalanine is an essential amino acid that plays a role in protein and neurotransmitter metabolism. During conditions of ischemia (lack of blood flow), an increase in phenylalanine levels is associated with the activation of proteolytic processes, mobilization of amino acid pools, and metabolic disturbances due to inhibition of the activity of phenylalanine hydroxylase, which occurs against a background of oxidative stress and inflammatory responses (7–9, 38). A further contribution may be provided by a systemic inflammatory reaction, accompanied by an elevation in the levels of pro-inflammatory cytokines (26, 27).

The results obtained are in line with the findings of metabolic studies, which consider phenylalanine to be a sensitive indicator of metabolic stress and the catabolic state in patients with AIS (6, 36, 37).

Therefore, an increase in phenylalanine concentration reflects the hyperacute phase of the systemic metabolic response to ischemia, but the changes observed in this hyperacute phase are primarily non-specific.

In the hyperacute phase of cerebrovascular events, intergroup variations in arginine levels were observed, with higher concentrations in TIA and lower levels in AIS. These findings reflect differences in the regulation of nitric oxide (NO)-dependent mechanisms and endothelial function, which are characteristic of these conditions.

Arginine is an essential precursor for the biosynthesis of NO, which regulates vascular tone and microcirculation. Under conditions of ischemia, a decrease in arginine levels may be associated with an increased demand, due to activation of inducible nitric oxide synthase (iNOS) and inflammatory processes. Additionally, an imbalance between iNOS and arginase activity may contribute to a reduction in arginine bioavailability (9, 39, 40).

Higher arginine levels in TIA may reflect the relative preservation of endothelial function and the lower severity of metabolic response.

According to the literature, changes in arginine levels during AIS are phase-dependent, and in later stages, its increase may be linked to the severity of the condition and an unfavourable outcome (36, 41).

Therefore, a decrease in arginine levels in the hyperacute phase following AIS is indicative of the activation of NO-dependent and inflammatory pathways, and can be regarded as an early marker of endothelial dysfunction.

During the hyperacute phase of acute cerebrovascular disease, a decrease in tryptophan levels was observed in all clinical groups when compared to the control group, with the most significant decrease seen in patients with TIA, reflecting an early activation of metabolic and neuroimmunological mechanisms.

Tryptophan is a vital amino acid whose metabolic process is closely connected to inflammation and oxidative stress. The reduction in its level during ischemia is attributed to the activation of the kynurenine metabolic pathway under the influence of pro-inflammatory cytokines and the enzyme indolamine-2,3-dioxygenase (10). Literature data support a reduction in tryptophan levels following AIS, albeit early alterations may be variable and accompanied by an elevation in the kynurenic acid/tryptophan ratio (10, 42–44).

Activation of the kynurenine pathway results in the formation of neuroactive metabolites, which can contribute to neuroinflammation and neuronal damage. This suggests that a decrease in tryptophan levels may be an indicator of neuroimmune activation.

A more significant decrease in tryptophan during TIA may reflect the sensitivity of this amino acid to early ischemic episodes and the intensity of systemic inflammation. Therefore, a reduction in tryptophan concentrations in the hyperacute phase is associated with the activation of the kynurenine pathway, confirming its role as a sensitive marker for early neuroimmune dysfunction in ischemic brain injury.

In general, the data obtained suggest that systematic changes in the amino acid profile occur already in the initial stages of the disease and reflect key aspects of the pathogenesis, such as energy deficiency, endothelial dysfunction, and neuroimmune activation. This finding supports the diagnostic importance of the amino acid panel at hyperacute phase of the condition.

### 4.5 Dynamic metabolite changes (*Δ* analysis)

Analysis of the dynamics of amino acid profiles has shown that metabolic changes during the early stages of acute cerebrovascular disease occur in phases and can be differentiated clinically. In contrast to assessing absolute values, analysis of *Δ*-indices reveals the direction and magnitude of metabolic alterations.

The most significant form-specific changes were observed for amino acids involved in energy metabolism and catabolism. Valine and phenylalanine showed directional dynamics, particularly pronounced in transient ischemic attacks, which may indicate the activation of adaptive mechanisms during reversible ischemia (6–8, 36). In the AIS group, a reduction in valine levels combined with an increase in arginine levels indicates a shift towards metabolic reorganization involving NO-dependent mechanisms and endothelial dysfunction (41).

Tryptophan demonstrated similar dynamics in all groups, indicating the systemic activation of neuroimmune processes associated with the kynurenine metabolic pathway (10, 45). At the same time, lysine exhibited intragroup dynamic patterns without intergroup variation, indicating its involvement in generalized metabolic reorganization without pronounced specificity (6, 36).

Glutamic acid did not exhibit significant changes, which may be attributed to the tight regulation of the glutamate-glutamine cycle.

**Figure 5:**
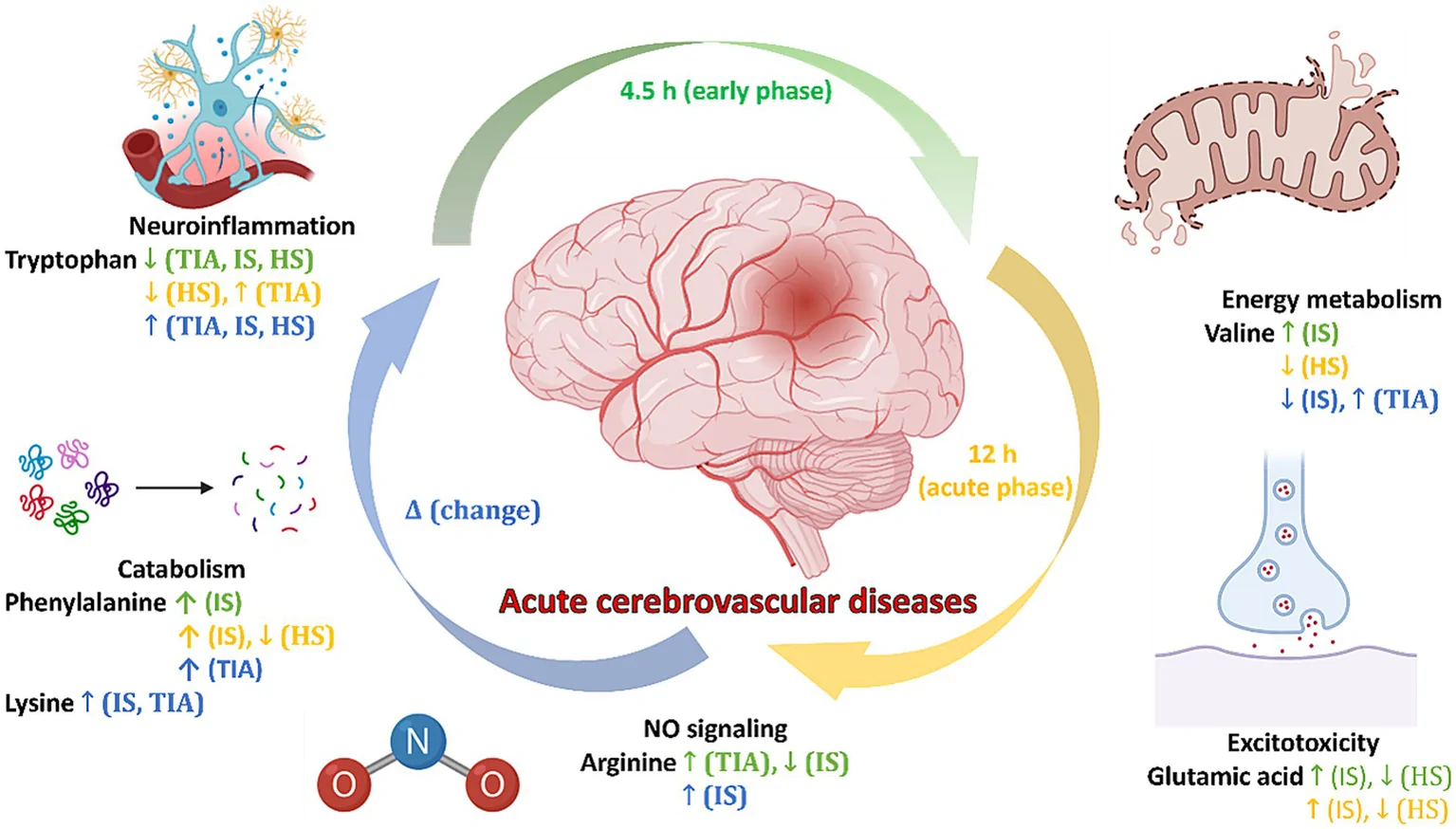
Integrative scheme of amino acid profile dynamics in acute cerebrovascular diseases. The diagram illustrates directional changes in plasma amino acid concentrations between the hyperacute phase (0–4.5 h) and 12 h after the onset of acute neurological symptoms. Key pathobiochemical mechanisms are represented, including neuroinflammation, energy metabolism, catabolism, nitric oxide-dependent processes, and excitotoxicity. Green indicates statistically significant differences in the hyperacute phase (0–4.5 h), yellow indicates the acute phase (12 h), and blue indicates the difference in metabolite concentrations between the first and second time points. Arrows (↑/↓/↔) indicate the direction of changes in metabolite concentrations over time.

Therefore, the metabolic response in acute cerebrovascular diseases is a complex and dynamic process, in which various amino acid levels reflect individual aspects of the pathogenesis (Figure 5). The evaluation of *Δ*-indices allows for a more accurate characterization of the direction of metabolic alterations and has a greater diagnostic value compared to the analysis of single time points alone.

### 4.6 Diagnostic potential of amino acid profile analysis

The findings suggest that the plasma amino acid profile in patients with acute cerebrovascular diseases is associated with a complex systemic metabolic response occurring during acute brain injury. The observed alterations in valine, phenylalanine, tryptophan, and glutamic acid are broadly consistent with previous evidence implicating amino acid metabolism in energy imbalance, excitotoxicity, and neuroinflammatory responses (6, 7). However, because these pathways were not directly assessed in the present study, the underlying mechanistic interpretations remain tentative.

Despite statistically significant between-group differences, individual amino acids demonstrated limited diagnostic utility when assessed in isolation. ROC analysis showed limited discriminative ability, with AUC values ranging approximately from 0.61 to 0.65, consistent with the concept that individual circulating metabolites are unlikely to provide sufficient diagnostic accuracy as standalone biomarkers (6, 46, 47).

In the initial extended multivariable model, phenylalanine and tryptophan remained independently associated with AIS after simultaneous evaluation with age, sex, leukocyte count, and glutamic acid. In the optimized model, the addition of phenylalanine and tryptophan to the clinical and laboratory variables increased the AUC from 0.734 to 0.803 (*p* = 0.007), indicating an incremental improvement in discrimination within the development cohort (7). These findings support the potential value of integrating metabolomic markers with routinely available clinical and laboratory variables rather than using individual amino acids in isolation.

Phenylalanine and tryptophan may be associated with systemic catabolic and inflammatory metabolic responses occurring during acute cerebral injury (7, 43). In particular, changes in tryptophan may be consistent with alterations in kynurenine-related metabolism; however, kynurenine and its downstream metabolites were not directly measured, and activation of this pathway cannot be confirmed from the present data.

The amino acid profile showed greater between-group differentiation during the hyperacute phase. At the second time point, fewer metabolites retained statistically significant between-group differences, which may reflect the temporal evolution of the systemic metabolic response, as well as the possible influence of early treatment and other clinical factors, rather than complete metabolic stabilization (48).

From a practical perspective, plasma amino acid profiling should not be considered a substitute for neuroimaging. Instead, it may provide complementary information regarding systemic metabolic and inflammatory responses, particularly during the limited therapeutic window (49–51). Therefore, the amino acid profile may have greater diagnostic value when interpreted together with clinical, laboratory, and neuroimaging findings (7).

Circulating metabolic and biochemical biomarkers may also have clinical applications beyond early diagnostic classification. Qiu et al. (52) investigated 914 patients with established intracerebral hemorrhage and found that higher serum total bilirubin levels were independently associated with an increased risk of 28-day all-cause mortality. These findings highlight the potential role of circulating biochemical markers in prognostic stratification after intracerebral hemorrhage. However, their study evaluated prognosis in patients with an already established diagnosis, whereas the present study focused primarily on the hyperacute diagnostic differentiation of AIS, HS, TIA, and control participants using plasma amino acid profiles. Therefore, diagnostic classification and prognostic outcome stratification should be considered distinct, although potentially complementary, applications of circulating biomarkers.

## 5 Limitations

Although a standardized HPLC–MS protocol and routine analytical quality-control procedures were applied throughout the study, the complete numerical analytical-validation dataset was not available for reporting in the present manuscript. In particular, numerical data on calibration ranges, limits of detection and quantification, accuracy, precision, recovery, and carryover could not be provided. This limitation should be considered when evaluating the analytical reproducibility and transferability of the reported measurements.

Although the first blood sample was collected before the initiation of acute treatment, some patients received standard stroke therapy, including intravenous thrombolysis, before the second blood sampling. In addition, fasting status, nutritional intake, intravenous fluid administration, and concomitant medications before the second sampling were neither standardized nor incorporated into the statistical analyses. These factors may have influenced plasma amino acid concentrations at the second time point and should therefore be considered when interpreting the observed temporal changes.

Several routine clinical and laboratory variables were incompletely available because they were extracted from medical records. Analyses involving these variables were therefore conducted using available observations, without imputation of missing values. Because the mechanism of missingness could not be formally assessed, the possibility of non-random missing data cannot be excluded, particularly in exploratory analyses involving routine laboratory parameters.

Although the multivariable diagnostic analyses accounted for selected demographic, clinical, and inflammatory covariates, the primary between-group comparisons of amino acid concentrations were not comprehensively adjusted for all potential confounders. Age, sex, neurological severity, diabetes mellitus, renal function, and systemic inflammatory status may independently influence circulating amino acid concentrations. Diabetes mellitus and renal-function parameters could not be consistently incorporated because of incomplete routine clinical data. Consequently, residual confounding cannot be excluded, and the observed metabolite differences should be interpreted as associations rather than evidence of disease-specific or causal metabolic mechanisms.

Finally, the diagnostic models reported in the present manuscript were developed and evaluated using the same study cohort. Model calibration and internal validation procedures, such as bootstrap resampling or cross-validation, were not performed for these models. Consequently, the reported discrimination estimates may be optimistic. External validation in independent and prospectively recruited cohorts is required before the proposed models can be considered for clinical implementation.

## 6 Conclusion

The amino acid profile of plasma in patients with acute cerebrovascular disease reflects the systemic metabolic response associated with ischemic injury and demonstrates specific changes that are most pronounced in the hyperacute phase.

The use of individual amino acids in isolation has limited diagnostic accuracy, confirming their role as part of interconnected metabolic processes rather than as independent biomarkers.

Analysis of dynamic changes in amino acid levels (*Δ*-indices) can identify additional patterns in the metabolic response and improve the differentiation between AIS and TIA compared to assessment at individual time points.

Integration of metabolic parameters with clinical data significantly improves the diagnostic accuracy of the model, indicating the added value of amino acid profiles in the early detection of AIS.

Therefore, the metabolomic approach may be considered a promising addition to standard clinical and instrumental diagnostic techniques for acute cerebrovascular conditions.

## Data Availability

The raw data supporting the conclusions of this article will be made available by the authors, without undue reservation.

## References

[1] GBD 2021 Demographics Collaborators. Global age-sex-specific mortality, life expectancy, and population estimates in 204 countries and territories and 811 subnational locations, 1950–2021, and the impact of the COVID-19 pandemic: a comprehensive demographic analysis for the Global Burden of Disease Study 2021. Lancet. (2024) 403:1989–2056. doi: 10.1016/S0140-6736(24)00476-8, 38484753 PMC11126395

[2] Collaborators GS. Global, regional, and national burden of stroke and its risk factors, 1990-2019: a systematic analysis for the global burden of disease study 2019. Lancet Neurol. (2021) 20:795–820. doi: 10.1016/S1474-4422(21)00252-0, 34487721 PMC8443449

[3] Buck BH Akhtar N Alrohimi A Khan K Shuaib A. Stroke mimics: incidence, aetiology, clinical features and treatment. Ann Med. (2021) 53:420–36. doi: 10.1080/07853890.2021.1890205, 33678099 PMC7939567

[4] Nukovic JJ Opancina V Ciceri E Muto M Zdravkovic N Altin A . Neuroimaging modalities used for ischemic stroke diagnosis and monitoring. Medicina (Kaunas). (2023) 59:1908. doi: 10.3390/medicina59111908, 38003957 PMC10673396

[5] Czap AL Sheth SA. Overview of imaging modalities in stroke. Neurology. (2021) 97:S42–51. doi: 10.1212/WNL.0000000000012794, 34785603 PMC11418094

[6] Wang XP Yan D Jin XP Zhang WY Shi T Wang X . The role of amino acid metabolism alterations in acute ischemic stroke: from mechanism to application. Pharmacol Res. (2024) 207:107313. doi: 10.1016/j.phrs.2024.107313, 39025169

[7] Ding L Zhang M Fan B Deng F Li Z Han Y . Metabolomics reveals key biomarkers for ischemic stroke: a systematic review of emerging evidence. Front Neurol. (2025) 16:1630390. doi: 10.3389/fneur.2025.1630390, 40860967 PMC12370529

[8] Licari C Tenori L Giusti B Sticchi E Kura A De Cario R Analysis of metabolite and lipid association networks reveals molecular mechanisms associated with 3-month mortality and poor functional outcomes in patients with acute ischemic stroke after thrombolytic treatment with recombinant tissue plasminogen activator. J Proteome Res (2021) 20:4758–4770. doi: 10.1021/acs.jproteome.1c00406 PMC8491161 34473513

[9] Khassafi N Azami Tameh A Mirzaei H Rafat A Barati S Vahidinia Z. Crosstalk between Nrf2 signaling pathway and inflammation in ischemic stroke: mechanisms of action and therapeutic implications. Exp Neurol. (2024) 373:114655. doi: 10.1016/j.expneurol.2023.114655, 38110142

[10] Hajsl M Hlavackova A Broulikova K Sramek M Maly M Dyr JE . Tryptophan metabolism, inflammation, and oxidative stress in patients with neurovascular disease. Meta. (2020) 10:208. doi: 10.3390/metabo10050208, 32438592 PMC7281607

[11] Grigolashvili M Klyuyev D Kasatkin D Battakova S Shayakhmetova Y Beisembayeva M . Evaluation of stroke care delivery: 12-month experience of a tertiary stroke center in Central Asia. Int J Clin Pract. (2025) 2025:8109173. doi: 10.1155/ijcp/8109173

[12] Sebastiani P Monti S Lustgarten MS Song Z Ellis D Tian Q . Metabolite signatures of chronological age, aging, survival, and longevity. Cell Rep. (2024) 43:114913. doi: 10.1016/j.celrep.2024.114913, 39504246 PMC11656345

[13] González-Beltrán D Yévenes-Briones H Lana A Cárdenas-Valladolid J Ángel Salinero-Fort M Rodríguez-Artalejo F . Prospective association between plasma amino acids and healthy aging in older adults. J Intern Med. (2025) 298:123–34. doi: 10.1111/joim.20105, 40501124 PMC12239062

[14] Castro A Signini É De Oliveira JM Di Medeiros Leal MCB Rehder-Santos P Millan-Mattos JC . The aging process: a metabolomics perspective. Molecules. (2022) 27:8656. doi: 10.3390/molecules27248656, 36557788 PMC9785117

[15] Bushnell CD Chaturvedi S Gage KR Herson PS Hurn PD Jiménez MC . Sex differences in stroke: challenges and opportunities. J Cereb Blood Flow Metab. (2018) 38:2179–91. doi: 10.1177/0271678X18793324, 30114967 PMC6282222

[16] Benjamin EJ Blaha MJ Chiuve SE Cushman M. Das SR Deo R. Heart disease and stroke statistics-2017 update: a report from the American Heart Association. Circulation (2017);135:e146–e603. doi: 10.1161/CIR.0000000000000485 PMC5408160 28122885

[17] Jang C Oh SF Wada S Rowe GC Liu L Chan MC . A branched-chain amino acid metabolite drives vascular fatty acid transport and causes insulin resistance. Nat Med. (2016) 22:421–6. doi: 10.1038/nm.4057, 26950361 PMC4949205

[18] Wiklund P Zhang X Pekkala S Autio R Kong L Yang Y . Insulin resistance is associated with altered amino acid metabolism and adipose tissue dysfunction in normoglycemic women. Sci Rep. (2016) 6:24540. doi: 10.1038/srep24540, 27080554 PMC4832240

[19] O'Donnell MJ Chin SL Rangarajan S Xavier D Liu L Zhang H . Global and regional effects of potentially modifiable risk factors associated with acute stroke in 32 countries (INTERSTROKE): a case-control study. Lancet. (2016) 388:761–75. doi: 10.1016/S0140-6736(16)30506-2, 27431356

[20] Feigin VL Brainin M Norrving B Martins SO Pandian J Lindsay P . World stroke organization: global stroke fact sheet 2025. Int J Stroke. (2025) 20:132–44. doi: 10.1177/17474930241308142, 39635884 PMC11786524

[21] Gallo G Volpe M Savoia C. Endothelial dysfunction in hypertension: current concepts and clinical implications. Front Med (Lausanne). (2021) 8:798958. doi: 10.3389/fmed.2021.798958 PMC8811286 35127755

[22] Amponsah-Offeh M Diaba-Nuhoho P Speier S Morawietz H. Oxidative stress, antioxidants and hypertension. Antioxidants (Basel). (2023) 12:281. doi: 10.3390/antiox12020281, 36829839 PMC9952760

[23] Al Ahmed F. Kennelly P. Herlihy D. Bejleri J. Williams D. J. Thornton J. J. Tissue is the issue: a systematic review of methods for the determination of infarct volume in acute ischaemic stroke. Brain Sci (2025);15:583. doi: 10.3390/brainsci15060583, 40563756. PMC12190565 40563756

[24] Saini V Guada L Yavagal DR. Global epidemiology of stroke and access to acute ischemic stroke interventions. Neurology. (2021) 97:S6–S16. doi: 10.1212/WNL.0000000000012781, 34785599

[25] Nikolakakis I Koutroulou I Mantatzis M Finitsis S Grigoriadis N Karapanayiotides T. Limitations and blind spots of diffusion-weighted imaging in the evaluation of acute brain ischemia: a narrative review. J Clin Med. (2026) 15:885. doi: 10.3390/jcm15020885, 41598822 PMC12842246

[26] Jickling GC Liu D Ander BP Stamova B Zhan X Sharp FR. Targeting neutrophils in ischemic stroke: translational insights from experimental studies. J Cereb Blood Flow Metab. (2015) 35:888–901. doi: 10.1038/jcbfm.2015.45, 25806703 PMC4640255

[27] Jayaraj RL Azimullah S Beiram R Jalal FY Rosenberg GA. Neuroinflammation: friend and foe for ischemic stroke. J Neuroinflammation. (2019) 16:142. doi: 10.1186/s12974-019-1516-2, 31291966 PMC6617684

[28] Wu F Liu Z Zhou L Ye D Zhu Y Huang K . Systemic immune responses after ischemic stroke: from the center to the periphery. Front Immunol. (2022) 13:911661. doi: 10.3389/fimmu.2022.911661, 36211352 PMC9533176

[29] Zhang J Ren Q Song Y He M Zeng Y Liu Z . Prognostic role of neutrophil-lymphocyte ratio in patients with acute ischemic stroke. Medicine (Baltimore). (2017) 96:e8624. doi: 10.1097/MD.0000000000008624, 29137097 PMC5690790

[30] Qun S Tang Y Sun J Liu Z Wu J Zhang J . Neutrophil-to-lymphocyte ratio predicts 3-month outcome of acute ischemic stroke. Neurotox Res. (2017) 31:444–52. doi: 10.1007/s12640-017-9707-z, 28181171

[31] Louveau A Harris TH Kipnis J. Revisiting the mechanisms of CNS immune privilege. Trends Immunol. (2015) 36:569–77. doi: 10.1016/j.it.2015.08.006, 26431936 PMC4593064

[32] Wang H Zhang S Xie L Zhong Z Yan F. Neuroinflammation and peripheral immunity: focus on ischemic stroke. Int Immunopharmacol. (2023) 120:110332. doi: 10.1016/j.intimp.2023.110332, 37253316

[33] Cui J Li H Chen Z Dong T He X Wei Y . Thrombo-inflammation and immunological response in ischemic stroke: focusing on platelet-Tregs interaction. Front Cell Neurosci. (2022) 16:955385. doi: 10.3389/fncel.2022.955385, 35846566 PMC9278516

[34] Yu Q Mao X Fu Z Luo S Huang Q Chen Q . Fasting blood glucose as a predictor of progressive infarction in men with acute ischemic stroke. J Int Med Res. (2022) 50:3000605221132416. doi: 10.1177/03000605221132416, 36271599 PMC9597044

[35] Campbell BCV Khatri P. Stroke. Lancet. (2020) 396:129–42. doi: 10.1016/S0140-6736(20)31179-X, 32653056

[36] Sidorov EV Rout M Xu C Jordan L Fields E Apple B . Difference in acute and chronic stage ischemic stroke metabolic markers with controls. J Stroke Cerebrovasc Dis. (2023) 32:107211. doi: 10.1016/j.jstrokecerebrovasdis.2023.107211, 37331250 PMC10527469

[37] Bein BN Ezhova LA. Izmenenie spektra svobodnykh aminokislot syvorotki krovi u bolnykh s tserebrovaskulyarnymi zabolevaniyami. Vyats Med Vestn. (2007) (2-3).

[38] Chen WS Wang CH Cheng CW Liu MH Chu CM Wu HP . Elevated plasma phenylalanine predicts mortality in critical patients with heart failure. ESC Heart Fail. (2020) 7:2884–93. doi: 10.1002/ehf2.12896, 32618142 PMC7524095

[39] Cinelli MA Do HT Miley GP Silverman RB. Inducible nitric oxide synthase: regulation, structure, and inhibition. Med Res Rev. (2020) 40:158–89. doi: 10.1002/med.21599, 31192483 PMC6908786

[40] Pourbagher-Shahri AM Farkhondeh T Talebi M Kopustinskiene DM Samarghandian S Bernatoniene J. An overview of NO signaling pathways in aging. Molecules. (2021) 26:4533. doi: 10.3390/molecules26154533, 34361685 PMC8348219

[41] Wang X Liu T Song H Cui S Liu G Christoforou A . Targeted metabolomic profiling reveals association between altered amino acids and poor functional recovery after stroke. Front Neurol. (2019) 10:1425. doi: 10.3389/fneur.2019.01425, 32082239 PMC7001531

[42] Saccaro LF Pico F Chadenat ML Richard O Launay JM Bastenaire B . Platelet, plasma, urinary tryptophan-serotonin-kynurenine axis markers in hyperacute brain ischemia patients: a prospective study. Front Neurol. (2021) 12:782317. doi: 10.3389/fneur.2021.782317, 35087467 PMC8787359

[43] Colpo GD Venna VR McCullough LD Teixeira AL. Systematic review on the involvement of the kynurenine pathway in stroke: pre-clinical and clinical evidence. Front Neurol. (2019) 10:778. doi: 10.3389/fneur.2019.00778, 31379727 PMC6659442

[44] Dylla L Higgins HM Poisson SN Vu T Reisz JA Herson PS . Sex differences in tryptophan metabolism via the kynurenine pathway in acute ischemic stroke. Clin Ther. (2024) 46:960–6. doi: 10.1016/j.clinthera.2024.10.015, 39603869 PMC11637908

[45] Yao C Xie D Zhang Y Shen Y Sun P Ma Z . Tryptophan metabolism and ischemic stroke: an intricate balance. Neural Regen Res. (2026) 21:466–77. doi: 10.4103/NRR.NRR-D-24-00777, 40326980 PMC12220693

[46] Tiedt S Brandmaier S Kollmeier H Duering M Artati A Adamski J . Circulating metabolites differentiate acute ischemic stroke from stroke mimics. Ann Neurol. (2020) 88:736–46. doi: 10.1002/ana.25859, 32748431

[47] Tao S Xiao X Li X Na F Na G Wang S . Targeted metabolomics reveals serum changes of amino acids in mild to moderate ischemic stroke and stroke mimics. Front Neurol. (2023) 14:1153193. doi: 10.3389/fneur.2023.1153193, 37122289 PMC10140586

[48] Iadecola C Buckwalter MS Anrather J. Immune responses to stroke: mechanisms, modulation, and therapeutic potential. J Clin Invest. (2020) 130:2777–88. doi: 10.1172/JCI135530, 32391806 PMC7260029

[49] Demeestere J Wouters A Christensen S Lemmens R Lansberg MG. Review of perfusion imaging in acute ischemic stroke: from time to tissue. Stroke. (2020) 51:1017–24. doi: 10.1161/strokeaha.119.028337, 32008460

[50] Bateman M Slater LA Leslie-Mazwi T Simonsen CZ Stuckey S Chandra RV. Diffusion and perfusion MR imaging in acute stroke: clinical utility and potential limitations for treatment selection. Top Magn Reson Imaging. (2017) 26:77–82. doi: 10.1097/RMR.0000000000000124, 28277459

[51] Wardlaw JM Keir SL Seymour J Lewis S Sandercock PA Dennis MS . What is the best imaging strategy for acute stroke? Health Technol Assess. (2004) 8:iii–ix-x, 1-180. doi: 10.3310/hta8010 14731377

[52] Qiu D Li G Dong Y. Association between serum total bilirubin levels and 28-day all-cause mortality after intracerebral hemorrhage. Front Neurol. (2025) 16:1529415. doi: 10.3389/fneur.2025.1529415, 40013002 PMC11860087

